# Climate change mitigation through dietary change: a systematic review of empirical and modelling studies on the environmental footprints and health effects of ‘sustainable diets’

**DOI:** 10.1088/1748-9326/abc2f7

**Published:** 2020-12-22

**Authors:** Stephanie Jarmul, Alan D Dangour, Rosemary Green, Zara Liew, Andy Haines, Pauline FD Scheelbeek

**Affiliations:** 1 Department of Population Health, London School of Hygiene & Tropical Medicine, London WC1E 7HT, United Kingdom; 2 Centre on Climate Change and Planetary Health, London School ofHygiene & Tropical Medicine, London WC1E 7HT, United Kingdom; 3 The Department of Public Health, Environments and Society, London School of Hygiene & Tropical Medicine, London WC1E 7HT, United Kingdom

**Keywords:** sustainable diets, health impacts, co-benefits, dietary change, greenhouse gas emissions, water use, land use

## Abstract

The adoption of healthy diets with low environmental impact has been widely promoted as an important climate change mitigation strategy. Typically, these diets are high in plant-sourced and low in animal-sourced and processed foods. Despite the fact that their environmental impacts vary, they are often referred to as ‘sustainable diets’. Here we systematically review the available published evidence on the effect of ‘sustainable diets’ on environmental footprints and human health. Eight databases (OvidSP-Medline, OvidSP-Embase, EBSCO-GreenFILE, Web of Science Core Collection, Scopus, OvidSP-CAB-Abstracts, OvidSP-AGRIS, and OvidSP-Global Health) were searched to identify literature (published 1999–2019) reporting health effects and environmental footprints of ‘sustainable diets’. Available evidence was mapped and pooled analysis was conducted by unique combinations of diet pattern, health and environmental outcome. Eighteen studies (412 measurements) met our inclusion criteria, distinguishing twelve non-mutually exclusive sustainable diet patterns, six environmental outcomes, and seven health outcomes. In 87% of measurements (n = 151) positive health outcomes were reported from ‘sustainable diets’ (average relative health improvement: 4.09% [95% CI −0.10–8.29]) when comparing ‘sustainable diets’ to current/baseline consumption patterns. Greenhouse gas emissions associated with ‘sustainable diets’ were on average 25.8%[95%CI −27.0 to −14.6] lower than current/baseline consumption patterns, with vegan diets reporting the largest reduction in GHG-emissions (−70.3% [95% CI: −90.2 to −50.4]), however, water use was frequently reported to be higher than current/baseline diets. Multiple benefits for both health and the environment were reported in the majority (n = 315[76%]) of measurements. We identified consistent evidence of both positive health effects and reduced environmental footprints accruing from ‘sustainable diets’. The notable exception of increased water use associated with ‘sustainable diets’ identifies that co-benefits are not universal and some trade-offs are likely. When carefully designed, evidence-based, and adapted to contextual factors, dietary change could play a pivotal role in climate change mitigation, sustainable food systems, and future population health.

## 1 Background

Major food system transformations are required as part of an integrated set of global actions to meet the Paris Agreement on climate [1] and multiple United Nations sustainable development goals (SDGs), including those on hunger (SDG 2), health (SDG 3), responsible production and consumption (SDG 12) and climate action (SDG 13) [2]. Globally 22% of children are stunted, 39% of adults overweight and 2 billion people anaemic (mainly due to iron deficiency) [3], and major transformations to the food system are needed to improve global health and ensure a sufficient supply of nutritious foods for all in the future. The global food system also has a major environmental footprint; it contributes 21%–37% of global greenhouse gas (GHG) emissions, and has a significant impact on land and water use and biodiversity [1, 4–6]. While technological advances have increased agricultural efficiency and reduced environmental footprints [7], the impact of food systems on the environment are expected to increase substantially by 2050, largely due to population growth and dietary change, particularly in rapidly transitioning economies [8, 9].

Multiple recent reports have promoted the adoption of diets with low environmental impact (or diets through which people aspire to consume more sustainably) as an important climate change mitigation strategy. Typically, these diets are high in plant-sourced foods and low in animal-sourced and processed foods. Despite the fact that their environmental impact—with respect to planetary boundaries—varies greatly, they are often referred to as ‘sustainable diets’ [9, 10]. The focus has been on adult diets because of the specific nutritional requirements for children. Analyses have typically highlighted the so-called ‘co-benefits’ for both population health and the environment of reduced consumption of animal-sourced food products (mainly red and processed meats and dairy) and increased consumption of plant-sourced foods [11, 12]. Reducing greenhouse gas emissions of food systems, along with other actions including major efforts to minimize food loss and waste, are also being widely promoted as likely to improve global health and also potentially result in economic benefits [13, 14]. The complexities of sustainable and healthy food systems reach far beyond these two dimensions, and include working conditions in the agricultural sector and animal welfare as well as cultural and socio-economic aspects of diets. The relationship between food systems and climate change is bi-directional, and climate change is currently affecting yields of crops and livestock products and is projected to continue to do so in the future [15, 16].

The evidence base on the health effects and environmental footprints of sustainable diets has grown rapidly over the past decade. Previous reviews have assessed the nutritional content of ‘sustainable diets’ [12, 17], their health effects or their environmental footprints [11, 18, 19]. Here we address a gap in evidence by systematically reviewing the published literature from empirical and modelling studies that assess both the environmental footprints and human health effects of ‘sustainable diets’— predominantly diets high in plant-sourced and low animal-sourced foods. We identify both co-benefits and co-harms that accrue from the numerous pathways through which diets affect health and impact the environment, to enable potential trade-offs to be considered and addressed in the decision-making processes. This review aims to assess the multipleimpacts on the environment and health of several forms of ‘sustainable diets’ in order to support the design of evidence-based climate change mitigation policy.

## 2 Methods

This review follows the Preferred Reporting Items for Systematic Reviews and Meta-Analyses (PRISMA) guidelines [20] and presents the ROSES flow-chart [21]. We searched eight literature databases (OvidSP Medline, OvidSP Embase, EBSCO GreenFILE, Web of Science Core Collection, Scopus, OvidSP CAB Abstracts, OvidSP AGRIS, and OvidSP Global Health) using the London School of Hygiene & Tropical Medicine institutional access and optimized our search strategy to identify studies that reported on both environmental and health outcomes of ‘sustainable diets’. Experts from the London School of Hygiene & Tropical Medicine library services were consulted and reviewed drafts of the search strings. We published our protocol and received peer-reviewed comments prior to initiating the database search [22]. Our search included terms to identify dietary change towards ‘sustainable diets’ (e.g. dietary shift, dietary change, sustainable consumption). Eight sources of grey literature were explored—through websites, reports and data repositories of CGIAR, Potsdam Institute for Climate Impact Research (PIK), Stockholm Environment Institute (SEI), FOLU-Systemiq, United Nations Social Development Network (UNSDN), International Institute for Applied Systems Analysis (IIASA), World Resource Institute (WRI), Food and Agricultural Organization of the United Nations (FAO)—to identify the need for an additional systematic search in grey literature. Our primary outcomes were changes in health including all-cause mortality and incidence of cancers, cardiovascular disease and diabetes, and the environment footprints of diets including GHG emissions, water footprints and land use. Search terms were first developed for the Web of Science Core Collection (appendix A) and adjusted accordingly to fit the search criteria for the other seven databases. Three experts in the field (based on personal knowledge) were contacted to review the final list of included papers and identify missing studies. Reference lists from included articles and relevant systematic reviews were examined to compliment the search strategy. Database searching was completed by one individual (SJ), title and abstract screening and full text reviews were completed manually and in duplicate (SJ and ZL), with adjudication as necessary by a third reviewer (PS). Data extraction was conducted in duplicate (SJ and PS).

### 2.1 Selection criteria

We included published empirical and modelling studies that reported the effect of dietary shifts, or comparison of different dietary patterns, and reported both health outcomes and environmental footprints. As scientific knowledge and the experimental and modelling rigour of environmental and health impact assessment has recently increased substantially, we only included evidence published over the past 20 years (between January 1999 and October 2019). We excluded review articles, articles with no quantitative outcomes and articles that did not meet quality criteria (see published protocol [22]). Study bias was assessed using criteria adapted from the Van Voorn checklist for modelling studies [23] and the CASP randomized control trial checklist [24].

### 2.2 Data analysis

Data were extracted, mapped and summarized in aggregates of dietary profile, health outcomes and environmental footprints. The dietary profile aggregate selection emerged from the identified studies and their respective author definitions. Geographic location of study setting was extracted and labelled as high-, middle-, or low-income country (HIC, MIC, LIC), using the World Bank classification [25]. Studies typically reported multiple ‘measurements’ with distinct combinations of exposures (diet), health effects and environmental footprints. We used these individual measurements reported within studies as the unit of analysis. We removed measurements that were duplicated within a single study or across multiple studies, with the exception of baseline measurements used to compare against several alternative diets. Measurements in which the exposure was ‘set’ (for example studies trying to identify what dietary change would be required for a certain % reduction in environmental footprint) were removed, as they performed post hoc modelling of diets under set conditions, rather than modelling environmental and health impacts of dietary change. Location and population age were recorded to assess potential differences by national income category (low, medium, high) and age (children, adults, older people).

For studies reporting data on ‘baseline’, ‘average’ or ‘business-as-usual’ health effects and environmental footprints (5 studies), these data were used as baseline values against which the health effects and environmental footprints of alternative diets were compared. For studies without baseline data, but for which relevant matched data on health and/or environmental footprints could be identified (e.g. reported by WHO or World Resources Institute) (13 Studies), the publicly available data were used as the comparator (appendix B).

The direction and relative difference (percentage change) in health and environmental outcomes were extracted for each individual measurement, comparing ‘sustainable diets’ to baseline diets. Outcome data were pooled, where a minimum of three studies— reporting on identical combinations of dietary shift, environmental outcome and health outcome—were available. Pooled analyses were adjusted for the nested nature of measurements within studies. For empirical studies, confidence intervals for pooled results were calculated, if sufficient studies (n = 3) reported on their individual confidence limits. For modelling studies, uncertainty estimates around pooled results were derived based on individually reported values, assuming additive uncertainty. When less than the required number of studies reported on a specific outcome uncertainty limits were still reported (for the reader’s information), but appear in *italics* in tables and figures. Sensitivity analysis was performed excluding studies that included adults >60 years of age and children as part of the study population, and excluding studies in low-income settings.

## 3 Results

### 3.1 Systematic search results

**Figure 1:**
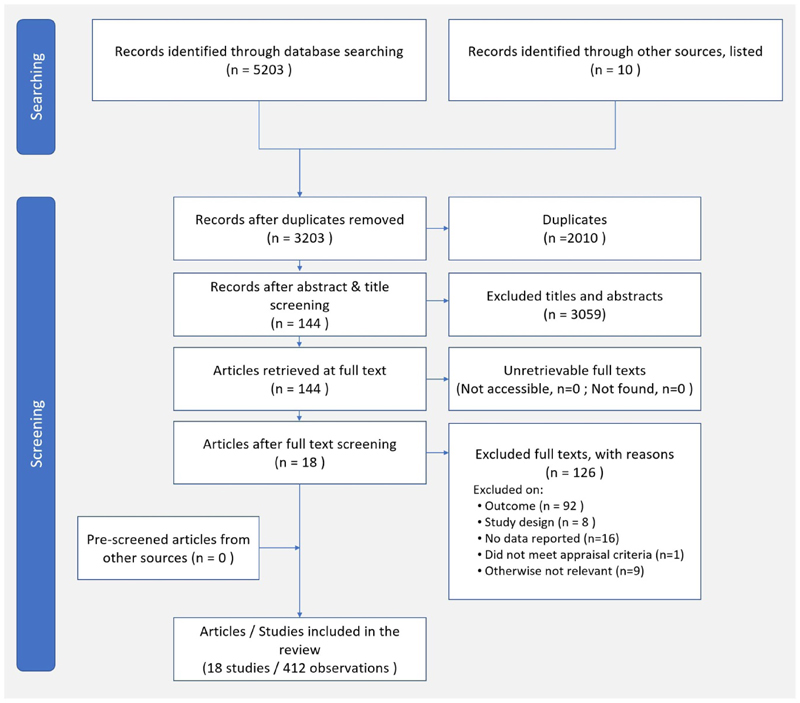
ROSES flow chart of searching, screening and inclusion of papers for systematic review on health effects and environmental footprints of ‘sustainable diets’.

**Figure 2:**
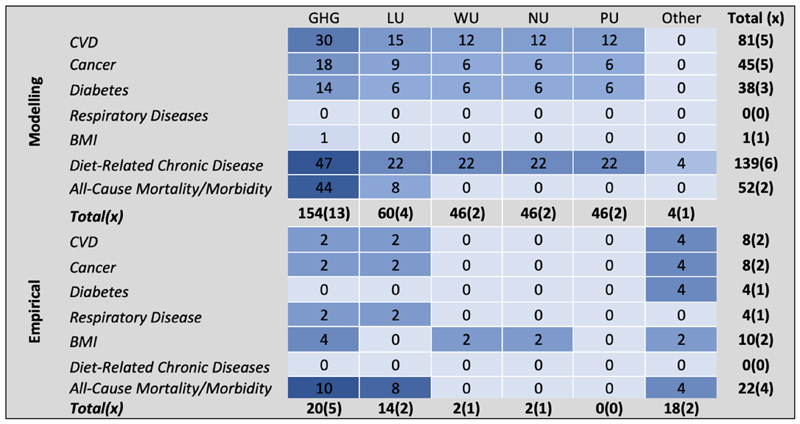
Heat map of health and environmental outcome combinations reported in 18 studies included in systematic review (values are number of measurements; (x) **=** number of studies); CVD **=** cardiovascular disease; ‘Diet-related Chronic Disease’ **=** Morbidity and/or mortality of combined nutrition related chronic diseases. GHG **=** Greenhouse Gas Emissions; LU **=** Land Use; WU **=** Water Use; NU **=** Nitrogen Use; PU **=** Phosphorus Use; Other includes acidification, biodiversity loss, and environmental footprint index.diets (+13.8% [95% CI: 8.72—18.9] in studies comparing baseline diets with those substituting ASF with PSF). There was generally good concordance in these findings across modelling and empirical studies.

Our initial search identified 3203 unique papers for title and abstract screening, and resulted in 144 articles eligible for full-text screening. Of these, 18 articles (13 modelling studies and 5 empirical studies) reporting 412 measurements, met our inclusion criteria (figure 1, figure 2, and appendix E). No further studies were identified through the grey literature exploration, hence no further systematic search of the grey literature was performed. The empirical studies included two cross-sectional and three longitudinal studies. A total of 412 measurements reported on unique health outcomes, with single or multiple corresponding environmental outcomes or unique environmental outcomes, with single or multiple corresponding health outcomes. Five studies (77 measurements) included all age groups, and thirteen studies (105 measurements) included only adults (3 among adults of all ages, 9 among adults aged 25-65 years, and 1 study among middle aged and older adults 45+ years). Fourteen studies were based on European data; other countries included Australia (1 study) and the United States (1 study), two studies categorized geographic location based on regions (i.e. South Asia, Sub-Saharan Africa) and incomerankings (high-, middle-, low-income) rather than specific countries. Two studies included scenarios for low-income countries.

Included studies examined a variety of overlapping dietary patterns (table 1) all of which incorporated a reduction in animal-sourced foods, particularly red and processed meats and dairy, and an increase in plant-sourced foods. For the purposes of further analyses the author-defined dietary patterns were categorised into 12 groups (table 1).

Seventeen studies (13 modelling studies; 4 empirical studies) reported estimated reductions in greenhouse gas emissions (in kg CO2 equivalents) associated with ‘sustainable diets’. Land, water, phosphorus and nitrogen use were also regularly reported (table 2), bringing the total number of identified environmental outcomes studied in the included papers to five. All-cause mortality, and combined mortality or morbidity of nutrition related chronic diseases (in DALYs, deaths averted, etc) were the most commonly reported health outcomes (8 modelling studies, 4 empirical studies), cardiovascular disease (CVD) (x = 7) and cancer (x = 7) were also frequently reported (table 2), totalling seven different identified health outcome aggregates.

### 3.2 Health impacts of ‘sustainable diets’

‘Sustainable diets’ were reported to improve health outcomes in 87% (n = 151) of the included measurements. In modelling studies, vegan diets and diets in which there was a degree of replacement of ASF with PSF had the largest effect on combined health outcomes (-13.9% [95% CI -22.7 to -5.2] and -7.3% [95% CI -11.1 to -3.6 respectively] that was broadly consistent across individual health outcomes. Author-defined ‘sustainable diets’ (labelled as ‘low GHG emission diet’) was the only category for which a significant worsening of health outcomes was identified (+12.8% [95% CI +8.78 to +16.9]), however this was based on a very small number (x = 2) of papers. Findings from modelling and empirical studies were typically not concordant.

### 3.3 Environmental impacts of ‘sustainable diets’

Studies evaluated a range of six different environmental outcomes associated with ‘sustainable diets’ (table 2 and appendix D) and consistently reported reductions in greenhouse gas emissions with the largest reduction associated with vegan (-81.4% [95% CI -87.7 to -75.1]), vegetarian (-74.6% [95% CI -82.3 to -66.8]), flexitarian (-46.9% [95% CI -55.2 to -38.5] and pescatarian (-46.5% [95% CI -83.4 to -9.54] diets. ‘Sustainable’ dietary patterns were also associated, in the majority of measurements, with reductions in land use (-8.93% [95% CI -18.1-0.29]) and nitrogen use (-11.2 [95% CI -18.0 to -4.45]). Water use was typically higher in ‘sustainable’ compared with baseline

### 3.4 Health effects and environmental footprints of ‘sustainable diets’

**Figure 3:**
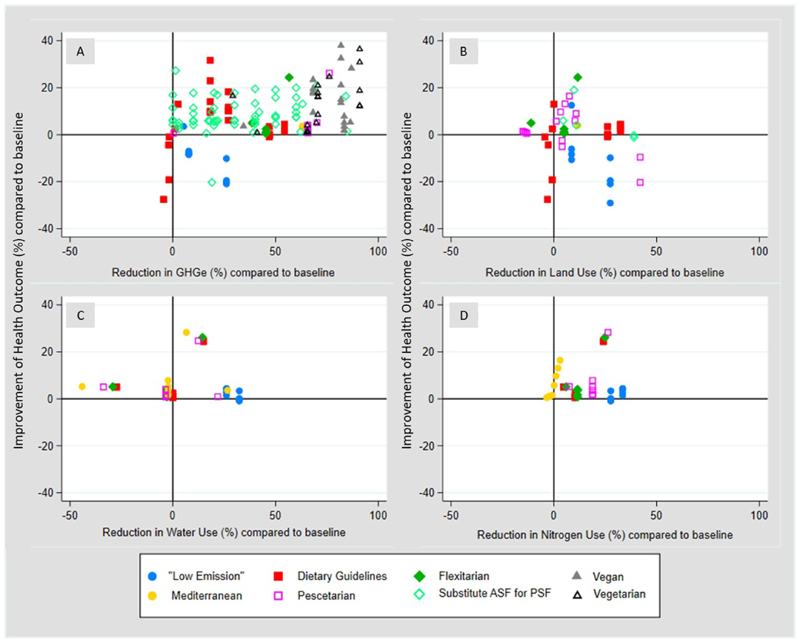
Reductions in (A) greenhouse gas emissions; (B) land use; (C) water use; and (4) nitrogen use; and their associated combined health outcomes for the eight most common dietary category reported in measurements included in systematic review. (GHGe **=** Greenhouse Gas Emissions; ASF **=** animal sourced foods; PSF **=** plant sourced foods.

Compared with baseline diets, ‘sustainable diets’ were associated with both positive health effects and reduced GHG emissions in the majority of reported measurements (n = 151[87%]) (figure 3); the remaining measurements reported increased GHG emissions (n = 1), negative health effects (n = 18), or both (n = 4). Vegan, flexitarian, pescatarian, and diets in which meat was substituted with other animal source foods consistently found positive health effects and reduced GHG emissions compared to baseline diets (figure 3(a)).

Sensitivity analysis was performed excluding studies with older adults (65+ years of age) or children (0-18 years of age) in their study populations and studies performed in low-income countries. These exclusions did not change the direction and scale of the relationship between dietary change and health and environmental outcomes, with a few differences in statistical significance of pooled findings. It should be noted that the available evidence on the excluded age groups and low-income settings was relatively sparse. Age-specific results can be found in the appendix C. Two studies included in our analysis [30, 31] assessed the differences in health outcomes of high-, middle-, and low-income countries from shifts toward ‘sustainable diets’ found that the greatest improvement in per capita risk reductions occurred in high- and middle-income countries. These improvements were primarily driven by reduced red meat consumption and increased fruit and vegetable consumption.

When looking at land and nitrogen use we see similar trends reported in the included studies: compared with baseline diets, shifts towards ‘sustainable diets’ were reported to reduce land use and improve health in the majority of measurements (n = 45 [61%]), with 8 measurements (11%) reporting health, but no land use benefits, 17 measurements reporting land use but no health benefits, and 4 (5.4%) reporting on harms for both health and land use. The later were all evaluating shifts to ‘guideline diets’ (figure 3(b)). Nitrogen use was reported to be reduced in 44 (92%) experiments of which 39 (81% of all experiments) also showed an improvement in health; four experiments (8.3%), all evaluating shifts towards pescatarian diets, were found to increase nitrogen use (figure 3(d)), whilst 5 experiments (10.4%) showed a decrease in nitrogen use, but a detrimental health impact of shifts towards ‘sustainable diets’.

The reported change in combined water use and health outcomes of shifts toward ‘sustainable diets’ showed a different picture: only 27% of all measurements (n = 13) report both health and water use benefits of shifts towards ‘sustainable diets’. Throughout all dietary change aggregates, water use is reported in 67% of the measurements (n = 32) to increase when shifting from baseline to ‘sustainable diets’. Three (out of 10) measurements evaluating shifts from baseline to dietary guidelines reported a reduction in water use, but also poorer health outcomes (figure 3(c)).

## 4 Discussion

### 4.1 Research findings

This systematic review identified consistent evidence across a large spectrum of modelling and empirical studies of both positive health effects and reduced environmental footprints (especially lower GHG emissions and nitrogen use) accruing from diets with low environmental impact (or diets through which people aspire to consume more sustainably). The notable exception of increased water use—and to a lesser extent land use—associated with ‘sustainable diets’ identifies that co-benefits are not universal and some trade-offs are likely. 25% of the world’s population currently live in countries that face ‘extremely high’ levels of water stress [45], and it is therefore crucial for sustainable food system planning to identify these and other trade-offs at national and/or sub-national level. Evidence for some dietary environmental footprints (such as phosphorus use, loss of biodiversity, acidification) was scarce. A small number of studies showed adverse impacts on health and environmental outcomes from ‘sustainable diets’, suggesting a major role for local contextual factors including agricultural practices, trade strategies and specific foods consumed. The vast majority of identified studies were conducted in high- and middle-income countries, and a clear gap in evidence from low-income settings was revealed by this review.

The review identified many definitions of ‘sustainable diets’, most of which showed benefits for both health and the environment. Given that population shifts in diets require large scale behaviour change, this flexibility in definition may be crucial as it creates the opportunity to develop bespoke dietary guidelines and recommendations that could represent more realistic changes from current diets and would thereby ensure a good fit for the contextual underlying population diet and health profile. There were notable differences in the results reported in modelling studies and the relatively small number of empirical studies. Whilst a few of the small number of empirical studies—included in this review—report on negative health impacts, a recent empirical study from the UK (including over 500 000 adults) showed that adherence to national dietary guidelines is associated with a statistically significant reduction in mortality and dietary GHG-emission as compared to average diets, whilst dietary water footprints were similar across aggregates of varying levels of adherence to the guidelines ([46]—this study was published after completion of the systematic database search). This underlines that real-world and context-specific validation of the health, environmental and broader effects of ‘sustainable diets’ remains important.

### 4.2 Interpretation & context

Whilst we consider two impact ‘dimensions’—health and environmental footprints—in this review, there are several other important dimensions to consider. For example, large-scale shifts away from animal sourced foods could ultimately substantially change (global) food prices with substantial impacts on animal welfare and the livelihoods of producers. In addition, there are important trade-offs between different environmental impacts for example substantially increased production and supply of plantbased foods could increase pressure on scarce land and water resources especially in major producing areas typically in low and middle-income countries. Further development of context-specific ‘Planetary Health Diets’ that would further contribute to efforts to stay within all nine planetary boundaries [47], would be crucial for protecting human health and the natural systems on which this depends.

Behavioural interventions to improve health and/or environmental sustainability often have the potential for substantial impact, however they have proved difficult to achieve, especially when immediate benefits, for health, well-being or economic situation, are not directly experienced (e.g. [48].). A strong policy framework that that supports dietary choices improves health and the environment would facilitate behaviour change at scale [49]. Such a framework should include an evidence-based selection of components, such as (and not restricted to) regulations for mandatory and enhanced food labelling to inform consumers on the footprints of purchased products, an extended curriculum on sustainable diets in secondary education, or increased budget allocation for sustainable cooking classes.

Diets that were vegan, vegetarian, pescatarian or followed national dietary guidelines typically showed the most profound impacts on both health outcomes and environmental footprints. Up to 19.3% reductions were reported for health outcomes such as diabetes (average effect - 4% for all health outcomes), and large average reductions reported for food system greenhouse gas emissions and land use (-24% and -9% respectively) and extreme greenhouse gas reductions of up to 80% associated with vegan diets. Comparing this to other interventions related to behaviour change—such as active commutes to work—shifts towards more ‘sustainable diets’ show similar positive health and environmental impacts: active commuting was, for example reported to lower risk of all-cause mortality by 8% and diabetes by 30% [50], whilst a study from Stockholm [51] reported a 7% reduction of nitrous oxides and black carbon in the most densely populated inner-city areas as result of active commuting interventions.

### 4.3 Strengths and limitations

To our knowledge this is the first systematic review and pooled analysis evaluating the published peer-reviewed evidence on coexisting health and environmental benefits from adoption of diets with a low environmental impact or through which people aspire to consume more sustainably. We used comprehensive and rigorous search strings to identify the largest possible number of peer-reviewed papers to systematically present the totality of the available evidence base. Our study was subject to a number of limitations. First, relying on literature databases of published papers will inevitably introduce reporting and publication bias. It is not possible to assess the scale and impact of these biases on the overall findings of this study, however—after exploring eight highly relevant sources of grey literature and finding no additional data or data sources for this review— we assume to have missed only a small volume of additional findings that would possibly have been identified if a systematic grey literature review would have been performed. Furthermore, heterogeneity of study designs, definitions of ‘sustainable diets’—even within our defined dietary categories, and study setting, limited the possibilities to pool results. Some of the dietary categories (partly) overlap and author definitions were sometimes poorly described, which further challenged the labelling of sustainable diet groups. Nonetheless, direction and scale of evidence on health and environmental impact of dietary shifts was relatively consistent across studies with various definitions of ‘sustainable diets’. Dietary environmental footprints were predominantly measured using life cycle assessment (LCA) methods: whilst this is generally regarded as the ‘gold standard’ for this type of estimation, the method comes with its limitations and uncertainties around the estimates it produces. Finally, the restricted geographical spread of study settings, and the low number of studies in low- and middle-income settings limited the possibilities of geographical analysis, and exploration of socioeconomic and contextual differences in health and environmental impacts of similar dietary shifts.

### 4.4 Policy implications

Our study results suggest substantial co-benefits to both health and the environment accruing from the adoption of ‘sustainable diets’ and support recommendations from the UNFCCC and others (e.g. [8, 9, 43, 52].) that the adoption of‘sustainable diets’ is a powerful climate change mitigation strategy. Given the large spectrum of different dietary shifts that could ultimately lead to health and environmental benefits, and the various trade-offs of each of them, it is extremely important to further translate sustainable dietary recommendations into national and sub-national food strategies—including food based dietary guidelines. It will be crucial to identify, acknowledge and adequately address those that will be advantaged and disadvantaged by such guidelines particularly if the dietary changes are unaffordable for the poor, as successful climate change mitigation through diets will require transformational societal change.

### 4.5 Conclusions

Widescale adoption of diets with a low environmental impact offer an important opportunity for both climate change mitigation and health benefits through the food system. There are many different ways that such shifts could be shaped: contex-tualization and exploring realistic behaviour change options will be crucial in developing and implementing impactful recommendations around ‘sustainable diets’. Trade-offs beyond health and environmental impact should be assessed and need to be studied in more depth, notably the water requirements of major shifts to more plant-based food consumption. It will be important to avoid unintended consequences of such dietary shifts, notably due to (increased) international trade, which could alter availability of certain foods in exporting countries. When carefully designed, based on the latest evidence, and adopted to contextual factors, true ‘Planetary Health Diets’ could play a pivotal role in climate change mitigation, sustainable food systems, and population health in the future.

## Supplementary Material

Supplementary Appendix

## Figures and Tables

**Table 1:** Author-defined dietary descriptions in 18 studies included in systematic review and combined categories used in analyses (n = number of measurements; DHD = Dutch Healthy Diet index; RSN = Swiss Society for Nutrition; GDG = Global Dietary Guidelines; HDI = Healthy Diet Indicator; DASH = Dietary Approaches to Stop Hypertension; GBD = Global Burden of Disease; ASF = animal source foods; PSF = plant source foods; SS = starchy staples); GHG = greenhouse gas.

Author definition of diet	Dietary label in this review	Reference	N
‘Sustainable Diet’	Low GHG Emission	Biesbroek 2014 [26] Irz 2016 [27]	10
Adherence to dietary guidelines: DHD, RSN, GDG, HDI, DASH and GBD	Dietary Guidelines	Biesbroek 2017 [28] Chen 2019 [29] Springmann 2016 [30]	26
‘Flexitarian’	Flexitarian	Chen 2019 [29] Springmann 2018 a [31]	7
Increased consumption of PSF	Increase PSF	Springmann 2018b [32]	1
‘Mediterranean diet’	Mediterranean	Farchi 2OI7[33] Fresan 2018 [34]	6
Pescatarian OR increase in fish consumption	Pescatarian/increase fish	Chen 2019 [29] Irz 2017 [35] Salazar 2019 [36] Springmann 2018 a [31]	8
Reduction of meat or other ASF, no substitution	Reduce ASF no substitute	Aston 2012 [37] Biesbroek 2014 [26] Hobbs 2019 [38] Irz 2016 [27] Irz 2017 [35] Springmann 2018b [32]	15
Reduction of ASF with substitution with PSF	Substitute ASF with PSF	Biesbroek 2014 [26] Cobiac 2019 [39] Irz 2016 [27] Irz 2017 [35] Milner 2015 [40] Scarborough 2012 [41] Soret 2014 [42] Springmann 2018 a [31] Visecchia 2012 [43]	61
Reduction of ASF with substitution with SS	Substitute ASF with SS	Biesbroek 2014 [26]	2
Reduction of meat with substitution with other ASF	Substitute meat with ASF	Biesbroek 2014 [26] Scarborough 2012 [41]	6
‘Vegan’	Vegan	Chen 2019 [29] Rosi2Ol7 [44] Springmann 2016 [30] Springmann 2018 a [31]	18
‘Vegetarian’	Vegetarian	Chen 2019 [29] Fresan 2018 [34] Rosi 2017 [44] Soret 2014 [42] Springmann 2016 [30] Springmann 2018 a [31]	23

**Table 2:** Relative effect (%) on health outcomes by dietary category [exposure versus baseline] in 18 studies included in systematic review (n = number of measurements; ASF = Animal Sourced Foods; PSF = Plant Sourced Foods; SS = Starchy Staples; * = single study; ¥ = duplicates removed (multiple environmental outcomes)) and Relative effect (%) on environmental footprint by dietary category [exposure versus baseline] in 18 studies included in systematic review (n = number of measurements; ASF = Animal Sourced Foods; PSF = Plant Sourced Foods; SS = Starchy Staples; * = single study; ¥ = duplicates removed (multiple health outcomes)).

Diet category	Type of study	Health Outcomes	n	% effect by health outcome [95% Cl]	n	% effect on combined health outcomes [95% cl]	Environmental footprint	n	% impact by environmental footprint [95% Cl]	n	% impact on combined environmental footprints [95% Cl]
“Low GHG Emission Diets”		Respiratory Disease	2*	14.1 [5.48 to 22.7]			Greenhouse Gas Emissions	2*	-16.9 [-29.6 to-4.21]		
	Empirical	CVD	2*	14.0 [4.31 to 23.6]	9*	12.8 [8.78 to 16.9]				4*	-17.5 [-26.6 to-8.33]
		Cancer	2*	9.31 [8.14 to 10.5]			Land Use	2*	-18.0 [-31.Ito -4.95]		
		All-cause mortality	2*	13.9 [6.15 to 21.7]							
	Modelling						-	-	-	2*	-7.40 [-10.3 to -4.49]
Dietary Guidelines	Empirical	--	--	--	6*	6.12 [-4.70 to 16.9]	Greenhouse Gas Emissions	6*	1.10[-0.74 to 2.93]	12*	1.52 [0.39-2.66]
							Land Use	6*	1.86 [0.71 to 3.21]		
		Nutrition Related Chronic Diseases	5	-4.61 [-9.04 to -0.18]			Greenhouse Gas Emissions	4	-36.6 [-56.0 to -17.2]		
	Modelling	Cancer	4	-7.37 [-16.7 to 1.94]	20	-8.16 [-16.9 to 0.54]	Land Use	6*	-29.3 [-33.6 to-24.9]	12	-32.3 [-37.5 to -27.0]
		Diabetes	4	-12.3 [-24.3 to -0.27]			Nitrogen Use	2*	-30.6 [-34.6 to -26.5]		
		Cardiovascular Disease	8	-8.29 [-17.2 to 0.60]			Phosphorus Use	2*	-31.3 [-35.2 to-27.3]		
							Water Use	2*	-29.2 [-33.5 to -24.9]		
Flexitarian	Modelling	Nutrition Related Chronic Diseases	3	1.10 [-0.74 to 2.93]			Greenhouse Gas Emissions	3	-46.9 [-55.2 to -38.5]		
		Cardiovascular Disease	2*	-1.75 [-2.65 to -0.84]	7	-7.06 [-16.3 to 2.16]	Land Use Nitrogen Use	3 3	-1.82 [-12.6 to 8.96] -13.0 [-22.3 to -3.80]	15	-12.9 [-23.7 to -2.14]
							Phosphorus Use Water Use	3 3	-6.95 [-20.7 to 6.78] 4.09[-15.7 to 23.9]		
Mediterranean	Empirical	--	--	--	4	-4.37 [-29.6 to 20.8]	--	--	--	2	-34.2 [-74.2 to 5.85]
	Modelling	--	--	-	2*	-3.50 [-3.78 to -3.22]					
Pescatarian /increase in fish	Modelling	Nutrition Related Chronic Diseases	4	-8.43 [-18.6 to 1.74]	8	-6.20 [-13.5 to 1.14]	Greenhouse Gas Emissions	4	-46.5 [-83.4 to-9.54]	16	-16.9 [-31.0 to-2.76]
			2*	-3.81 [-4.15 to -3.48]			Land Use	3	-3.14 [-13.9 to 7.62]		
		Cardiovascular Disease					Nitrogen Use	3	-14.1 [-23.1 to 5.12]		
							Phosphorus Use	3	-7.81 [-21.5 to5.87]		
							Water Use	3	4.09 [-15.7 to 23.9]		
Reduce ASF - no Nutrition substitute	Empirical	-	-	-	2*	1.82 [-2.42 to 5.42]	-	-	-	3*	0.24 [-3.35 to 3.85]
		Nutrition Related Chronic Diseases	6	-0.26 [-0.40 to -0.14]			Greenhouse Gas Emissions	9	-6.07 [-10.7 to -1.44]		
	Modelling	Cancer	2*	-9.95 [-13.1 to -6.83]	13	-2.91 [-6.20 to 0.39]				12	-6.34 [-10.9 to -1.81]
		Diabetes	2*	-9.75 [-12.9 to -6.63]			Sulphur Dioxide	2	-1.75 [-2.79 to -0.71]		
		Cardiovascular Disease	2*	-8.05 [-10.3 to -5.76]							
Substitute ASF with PSF	Modelling	Nutrition Related Chronic	17	-6.01 [-7.96 to -4.06]			Greenhouse Gas Emissions	18	-18.6 [-34.4 to -2.79]		
		Cancer	2*	-6.05 [-11.0 to -1.13]			Land Use	12	-7.43 [-21.1 to 6.23]		
		Cardiovascular Disease	2*	7.60 [-10.0 to 25.2]	61	-7.33 [-11.1 to -3.59]	Nitrogen Use	8*	0.31 [-1.16 to 1.78]	55	-7.84 [-14.0 to -1.73]
		All-cause mortality	38	-10.2 [-11.9 to -8.45]			Phosphorus Use Water Use	8* 8*	0.81 [-1.22 to 2.83] 13.8 [8.72 to 18.9]		
Substitute meat with other SS	Modelling	--	--	--	2*	-5.75 [-13.0 to 1.52]	Greenhouse Gas Emissions Land Use	2* 2*	-10.5 [-10.9 to -9.96] -10.5 [-11.1 to -9.87]	4*	-10.5 [-11.1 to -9.87]
Substitute meat with other ASF	Modelling	All-cause mortality	3*	-9.67 [-17.2 to -2.14]	6	-4.93 [-10.4 to 0.54]	Greenhouse Gas Emissions Land Use	4 4	-3.85 [-7.69 to -0.01] -23.4 [-44.6 to -2.25]	8	-15.2 [-34.1 to 3.73]
Vegan	Empirical	Nutrition Related Chronic Diseases	5	-12.0 [-19.8 to -4.28]			-- Greenhouse Gas Emissions	-- 5	-- -81.4 [-87.7 to -75.1]	2*	-30.4 [-35.9 to -25.1]
	Modelling	Cancer	3	-11.7 [-23.4 to 0.06]	18	-13.9 [-22.7 to -5.20]	Land Use	3	-2.64 [-16.4 to 11.1]	17	-27.1 [-45.4to -8.80]
		Diabetes	3	-19.3 [-36.1 to -2.54]			Nitrogen Use	3	-17.6 [-26.3 to -8.81]		
		Cardiovascular Disease	6	-15.1 [-26.3 to -3.93]			Phosphorus Use Water Use	3 3	-11.2 [-25.7 to 3.36] 13.3 [-11.7 to 38.3]		
Vegetarian	Empirical	All-cause mortality	2*	7.82 [-25.9 to 41.5]	6	21.0 [-7.28 to 49.4]	Greenhouse Gas Emissions	2*	-35.1 [-43.3 to -26.9]	4	-25.8 [-36.4 to -15.2]
	Modelling	Nutrition Related Chronic Diseases	5	-10.4 [-17.4 to 3.37]			Greenhouse Gas Emissions	5	-74.6 [-82.3 to -66.8]		
		Cancer Diabetes Cardiovascular Disease	3 3 6	-10.1 [-20.6 to 0.50] -17.8 [-34.3 to -1.29] -12.3 [-24.8 to 0.16]	17	-11.9 [-20.8 to -3.00]	Land Use Nitrogen Use Phosphorus Use Water Use	3 3 3	-0.98 [-13.6 to 11.6] -14.5 [-24.2 to -4.86] -8.54 [-22.6 to 5.51] 8.31 [-13.3 to 30.0]	17	-30.2 [-55.0 to -5.42]
											
All diets	Combined*	--	--	--	182	-4.09 [-8.29 to 0.10]	Greenhouse Gas Emissions	70	-23.9 [-35.8 to -12.0]	--	--
							Water Use	41	-8.93 [-18.1 to 0.29]		
							Land Use	23	3.45 [-12.9 to 19.8]		
							Nitrogen Use	23	-11.2 [-18.0 to -4.45]		

## Data Availability

The data that support the findings of this study are available upon reasonable request from the authors.
