# Supplementary material for: Climate change mitigation through dietary change: a systematic review of empirical and modelling studies on the environmental footprints and health effects of ‘sustainable diets’
Source: Environ Res Lett. Author manuscript; Available in PMC 2021 Apr 22. (PMC7610659; doi:10.1088/1748-9326/abc2f7)
Supplement: Supplementary Appendix [file EMS120901-supplement-Supplementary_Appendix.pdf]

Appendix A

Table A1. Search strategy for web of science core collection.

Search #	Search term
#20	#13 AND #14 AND #19
#19	#15 OR #16 OR #17 OR #18
#18	TS = ((diet* OR consum* OR 'eating pattern' OR meal* OR nourish*) near/3 (current OR average* OR change* OR shift* OR choice* OR scenario* OR habit* OR sustain*))
#17	TS = ((plant-based OR fruit* OR vegetable* OR legume* OR nut* OR pulse*) near/3 (iodiver* OR higher))
#16	TS = ((meat OR animal-sourced OR dairy OR ultra-processed OR UPF) near/3 (reduc* OR decreas* OR free))
#15	TS = (vegan* OR vegetarian* OR flexitarian* OR pescatarian* OR sea-food OR seafood OR fish*)
#14	TS = ((climate OR environment*) near/5 (friendly OR footprint OR foot-print OR 'foot print' OR impact* OR damage* OR greenhouse OR land* OR 'land use' OR water* OR use* OR benefit* OR implication* OR carbon* OR sustain* OR iodiverse* OR nitrogen))
#13	#11 AND #12
#12	#3 OR #4 OR #5 OR #6 OR #7 OR #8 OR #9 OR #10
#11	#1 OR #2
#10	TS = (CKD OR cardiovascular OR cardio-vascular OR cancer OR BP)
#9	TS = 'kidney disease'
#8	TS = 'heart disease'
# 7	TS = (hypertension OR stroke OR diabetes OR ICH OR chronic)
# 6	TS = 'blood pressure'
#5	TS = (anemia OR anaemia)
# 4	TS = ((nutrient OR iron OR iodine OR 'vitamin D' OR 'vitamin B12' OR calcium OR 'Vitamin A' OR zinc OR magnesium) near/2 (deficien* OR shortage* OR value*))
# 3	TS = (obesity OR overweight OR over-weight OR underweight OR under-weight OR malnutrition OR malnour*)
# 2	TS = (prevalence OR incidence OR risk OR rate OR mortality or morbidity)
# 1	TS = (health* OR wellbeing OR well-being)

Appendix B. Sensitivity analysis health outcomes

Adults aged 25–70 only.

Diet Assessed	n	Type of study	Combined Health Impact (%) [95% CI]
"Sustainable Diets"	9*	Empirical studies	*12.8 [8.78 to 16.9]*
Dietary Guidelines	6*	Empirical studies	*6.12 [-4.70 to 16.9]*
Mediterranean	4	Empirical Studies	*-4.37 [-29.6 to 20.8]*
	2*	Modelling studies	*-3.50 [-3.78 to -3.22]*
Pescatarian /increase in fish	8	Modelling studies	*-6.20 [-13.5 to 1.14]*
Reduce ASF - no substitute	2*	Empirical studies	*1.82 [-2.42 to 5.42]*
	11	Modelling studies	*-3.62 [-7.43 to 0.19]*
Substitute ASF with PSF	8	Modelling studies	-2.44 [-8.61 to 3.74]
Substitute ASF with SS	2*	Modelling studies	-5.75 [-13.0 to 1.52]
Substitute meat with other ASF	6	Modelling studies	-4.93 [-10.4 to 0.54]

High- and middle-income countries only.

Diet Assessed	n	Type of study	Combined Health Impact (%) [95% CI]
"Sustainable Diets"	9*	Empirical studies	12.8 [8.78 to 16.9]
Dietary Guidelines	6*	Empirical studies	6.12 [-4.70 to 16.9]
	15	Modelling studies	-9.55 [-20.4 to 1.34]
Flexitarian	6	Modelling studies	-12.8 to [-28.9 to 3.34]
Mediterranean	4	Empirical Studies	-4.37 [-29.6 to 20.8]
	2*	Modelling studies	-3.50 [-3.78 to -3.22]
Pescatarian /increase in fish	7	Modelling studies	-9.64 [-22.8 to 3.54]
Reduce ASF - no substitute	2*	Empirical studies	1.82 [-2.42 to 5.42]
	13	Modelling studies	-2.91 [-6.20 to 0.39]
Substitute ASF with PSF	56	Modelling studies	-8.18 [-12.4 to -3.99]
Substitute ASF with SS	2*	Modelling studies	-5.75 [-13.0 to 1.52]
Substitute meat with other ASF	6	Modelling studies	-4.93 [-10.4 to 0.54]
Vegan	11	Modelling studies	-17.6 [-29.8 to -5.44]
Vegetarian	6	Empirical Studies	21.0 [-7.28 to 49.4]
	11	Modelling studies	-15.2 [27.2 to 3.11]

Appendix C. Origin of baseline data

Overview of source of baseline data of studies included in the review.

Author	Year of study	Baseline outcome source
Aston	2012	Paper
Biesbroek	2014	Paper
Biesbroek	2017	Paper
Chen	2019	Global burden of Disease
Cobiac	2019	Global burden of Disease
Farchi	2017	Paper
Hobbs	2019	Paper
Irz	2016	Paper
Irz	2017	Paper
Irz	2017	Paper
Milner	2015	Contacted Author
Rosi	2017	Paper
Scarborough	2012	Paper
Soret	2014	Paper
Springmann	2016	Paper
Springmann	2018a	Global burden of Disease
Springmann	2018b	Global Burden of Disease
Visecchia	2012	Paper

Appendix D

Figures D1(a) and D2(a) depict the range of relative difference in GHG emissions and land use reported in each scenario when shifting from baseline consumption patterns to sustainable diets. GHG emissions were consistently found to be inversely associated with shifts towards more sustainable diets

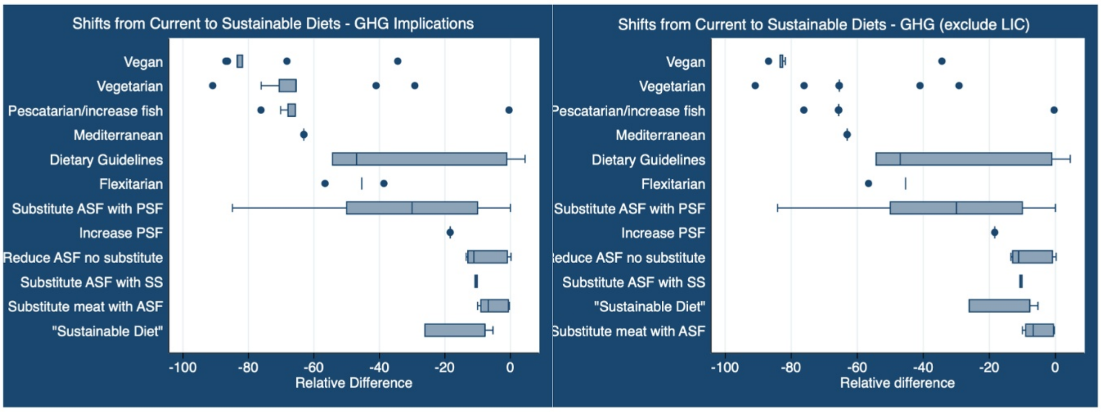

Figure D1. (a) Relative difference in GHG emissions when shifting from current to sustainable diets for all modelling studies and (b) Relative difference in GHG emissions when shifting from current to sustainable diets for middle- and high-income countries only.

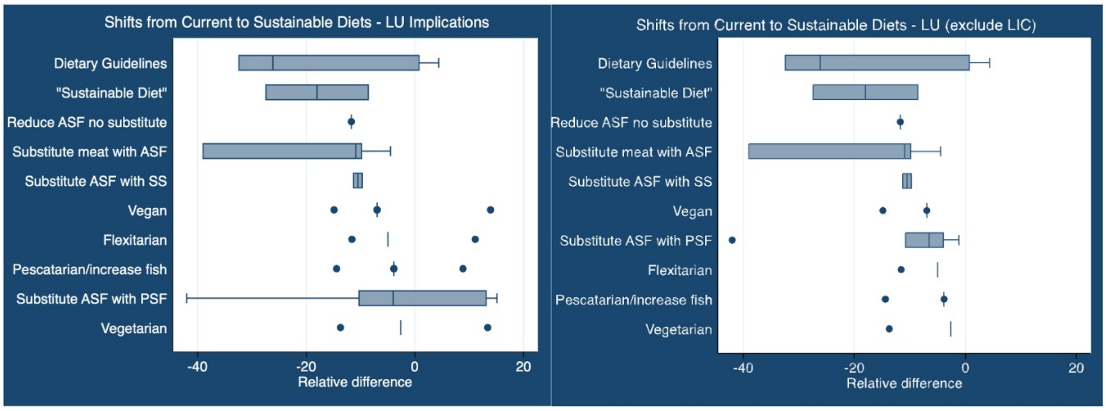

Figure D2. (a) Relative difference in Land Use when shifting from current to sustainable diets for all studies and (b) Relative difference in Land Use when shifting from current to sustainable diets for middle- and high-income countries only.

in each study, while the association with land use projections were inconclusive.

Sensitivity analysis excluding results from low-income countries (two studies) (figures D1(b) and D2(b)) show a reduction in both GHG and land use from shifts toward sustainable diets in middle- and high-income countries.

The largest reduction in GHG emissions were reported in studies assessing shifts to vegan diets (−70.3% [95% CI: −90.2 to −50.4]), vegetarian diets (−59.3% [95% CI: −76.0 to −42.5]), pescatarian diets (−46.4 [95% CI: −83.4 to −9.49], flexitarian diets (−46.0 [95% CI −49.6 to −42.4], and diets where animal source foods were replace by plant source foods (−25.5% [95% CI: −36.1 to −13.0]). Shift to dietary guidelines—that were not specifically aiming at a reduction in environmental footprints—showed to reduce GHG emissions on average by 24.1% (95% CI −48.0 to −0.15).

From a land use perspective, substituting animal sourced foods with plant sourced foods, shifting to 'Sustainable Diets', and adherence to various dietary guidelines were reported to be associated with the largest average reduction in land use (−23.7% [95% CI: −44.9 to −2.45]; −18.0% [95% CI: −24.5 to −11.5]; and −13.7% [95% CI: −35.3–7.97] respectively). Sensitivity analysis—excluding studies from low-income countries—did not significantly alter the results of the core analysis.

Combining evidence on water use of dietary shift showed statistically significant impacts for shifts towards two dietary patterns: shifting from current to dietary guidelines showed to reduce water footprints by 29.2% [95% CI −31.1 to −27.3], whilst dietary shifts whereby by animals sourced foods are replaced by plant sourced foods showed—on average an increase of 13.8% [95% CI 8.72–18.92], however both estimates were based on multiple measurements in a single study. All other dietary shifts did not show statistically significant impacts on water use.

Results on other environmental parameters—including change in use of phosphorus and nitrogen due to dietary shifts—can be found in appendix E.

Appendix E. Data included studies

Study number	Type of study	First author	Year of publication	Region	Final diet category	Health category	Health unit	Health—absolute difference	Health—relative difference	Env outcome	Env unit	Environment—absolute difference	Environment—relative difference
1	Modelling	Aston	2012	HIC/MIC	Reduce ASF no substitute	Cancer	% risk change	−12.20	−12.20	GHG	kg CO2e	−0.62	−13.48
1	Modelling	Aston	2012	HIC/MIC	Reduce ASF no substitute	Cancer	% risk change	−7.70	−7.70	GHG	kg CO2e	−0.43	−12.94
1	Modelling	Aston	2012	HIC/MIC	Reduce ASF no substitute	CVD	% risk change	−9.70	−9.70	GHG	kg CO2e	−0.62	−13.48
1	Modelling	Aston	2012	HIC/MIC	Reduce ASF no substitute	CVD	% risk change	−6.40	−6.40	GHG	kg CO2e	−0.43	−12.94
1	Modelling	Aston	2012	HIC/MIC	Reduce ASF no substitute	Diabetes	% risk change	−12.00	−12.00	GHG	kg CO2e	−0.62	−13.48
1	Modelling	Aston	2012	HIC/MIC	Reduce ASF no substitute	Diabetes	% risk change	−7.50	−7.50	GHG	kg CO2e	−0.43	−12.94
2	Empirical	Biesbroek	2014	HIC/MIC	'Sustainable Diet'	All-cause Mortality/Morbidity	deaths per 100000PY	36.93	8.31	GHG	kgCO2e/d	−0.30	−7.75
2	Empirical	Biesbroek	2014	HIC/MIC	'Sustainable Diet'	All-cause Mortality/Morbidity	deaths per 100000PY	86.83	19.54	GHG	kgCO2e/d	−1.01	−26.10
2	Empirical	Biesbroek	2014	HIC/MIC	'Sustainable Diet'	Cancer	deaths per 100000PY	18.15	8.47	GHG	kgCO2e/d	−0.30	−7.75
2	Empirical	Biesbroek	2014	HIC/MIC	'Sustainable Diet'	Cancer	deaths per 100000PY	21.76	10.16	GHG	kgCO2e/d	−1.01	−26.10
2	Empirical	Biesbroek	2014	HIC/MIC	'Sustainable Diet'	CVD	deaths per 100000PY	6.85	7.00	GHG	kgCO2e/d	−0.30	−7.75
2	Empirical	Biesbroek	2014	HIC/MIC	'Sustainable Diet'	CVD	deaths per 100000PY	20.49	20.93	GHG	kgCO2e/d	−1.01	−26.10
2	Empirical	Biesbroek	2014	HIC/MIC	'Sustainable Diet'	RD	deaths per 100000PY	1.94	7.87	GHG	kgCO2e/d	−0.30	−7.75
2	Empirical	Biesbroek	2014	HIC/MIC	'Sustainable Diet'	RD	deaths per 100000PY	4.99	20.27	GHG	kgCO2e/d	−1.01	−26.10
2	Empirical	Biesbroek	2014	HIC/MIC	'Sustainable Diet'	All-cause Mortality/Morbidity	deaths per 100000PY	36.93	8.31	LU	m2*year/d	−0.31	−8.59
2	Empirical	Biesbroek	2014	HIC/MIC	'Sustainable Diet'	All-cause Mortality/Morbidity	deaths per 100000PY	86.83	19.54	LU	m2*year/d	−0.99	−27.42

(Continued)

(continued).

Study number	Type of study	First author	Year of publication	Region	Final diet category	Health category	Health unit	Health—absolute difference	Health—relative difference	Env outcome	Env unit	Environment—absolute difference	Environment—relative difference
2	Empirical	Biesbroek	2014	HIC/MIC	'Sustainable Diet'	Cancer	deaths per 100000PY	13.08	6.10	LU	m2*year/d	−0.31	−8.59
2	Empirical	Biesbroek	2014	HIC/MIC	'Sustainable Diet'	Cancer	deaths per 100000PY	21.06	9.83	LU	m2*year/d	−0.99	−27.42
2	Empirical	Biesbroek	2014	HIC/MIC	'Sustainable Diet'	CVD	deaths per 100000PY	10.41	10.64	LU	m2*year/d	−0.31	−8.59
2	Empirical	Biesbroek	2014	HIC/MIC	'Sustainable Diet'	CVD	deaths per 100000PY	20.50	20.95	LU	m2*year/d	−0.99	−27.42
2	Empirical	Biesbroek	2014	HIC/MIC	'Sustainable Diet'	RD	deaths per 100000PY	−3.09	−12.56	LU	m2*year/d	−0.31	−8.59
2	Empirical	Biesbroek	2014	HIC/MIC	'Sustainable Diet'	RD	deaths per 100000PY	7.16	29.09	LU	m2*year/d	−0.99	−27.42
2	Modelling	Biesbroek	2014	HIC/MIC	Reduce ASF no substitute	All-cause Mortality/Morbidity	% Reduction		−4.00	GHG	% reduction		−11.50
2	Modelling	Biesbroek	2014	HIC/MIC	Substitute ASF with PSF	All-cause Mortality/Morbidity	% Reduction		−9.00	GHG	% reduction		−10.00
2	Modelling	Biesbroek	2014	HIC/MIC	Substitute ASF with PSF	All-cause Mortality/Morbidity	% Reduction		−6.00	GHG	% reduction		−10.00
2	Modelling	Biesbroek	2014	HIC/MIC	Substitute ASF with starchy staples	All-cause Mortality/Morbidity	% Reduction		−0.50	GHG	% reduction		−10.80
2	Modelling	Biesbroek	2014	HIC/MIC	Substitute ASF with starchy staples	All-cause Mortality/Morbidity	% Reduction		−11.00	GHG	% reduction		−10.10
2	Modelling	Biesbroek	2014	HIC/MIC	Substitute meat with other ASF	All-cause Mortality/Morbidity	% Reduction		−4.00	GHG	% reduction		−10.00
2	Modelling	Biesbroek	2014	HIC/MIC	Substitute meat with other ASF	All-cause Mortality/Morbidity	% Reduction		−19.00	GHG	% reduction		−4.50
2	Modelling	Biesbroek	2014	HIC/MIC	Substitute meat with other ASF	All-cause Mortality/Morbidity	% Reduction		−6.00	GHG	% reduction		−0.60

(Continued)

(continued).

Study number	Type of study	First author	Year of publication	Region	Final diet category	Health category	Health unit	Health—absolute difference	Health—relative difference	Env outcome	Env unit	Environment—absolute difference	Environment—relative difference
2	Modelling	Biesbroek	2014	HIC/MIC	Substitute ASF with starchy staples	All-cause Mortality/Morbidity	% Reduction		−0.50	LU	% reduction		−11.30
2	Modelling	Biesbroek	2014	HIC/MIC	Substitute ASF with starchy staples	All-cause Mortality/Morbidity	% Reduction		−11.00	LU	% reduction		−9.70
2	Modelling	Biesbroek	2014	HIC/MIC	Substitute ASF with PSF	All-cause Mortality/Morbidity	% Reduction		−9.00	LU	% reduction		−10.80
2	Modelling	Biesbroek	2014	HIC/MIC	Substitute ASF with PSF	All-cause Mortality/Morbidity	% Reduction		−6.00	LU	% reduction		−10.30
2	Modelling	Biesbroek	2014	HIC/MIC	Substitute meat with other ASF	All-cause Mortality/Morbidity	% Reduction		−4.00	LU	% reduction		−10.90
2	Modelling	Biesbroek	2014	HIC/MIC	Substitute meat with other ASF	All-cause Mortality/Morbidity	% Reduction		−19.00	LU	% reduction		−9.80
2	Modelling	Biesbroek	2014	HIC/MIC	Substitute meat with other ASF	All-cause Mortality/Morbidity	% Reduction		−6.00	LU	% reduction		−4.50
2	Modelling	Biesbroek	2014	HIC/MIC	Reduce ASF no substitute	All-cause Mortality/Morbidity	% Reduction		−4.00	LU	% reduction		−11.70
3	Empirical	Biesbroek	2017	HIC/MIC	Dietary Guidelines	All-cause Mortality/Morbidity	deaths per 100000PY	138.62	27.54	GHG	kgCO2e/d	0.21	4.49
3	Empirical	Biesbroek	2017	HIC/MIC	Dietary Guidelines	All-cause Mortality/Morbidity	deaths per 100000PY	−12.20	−2.42	GHG	kgCO2e/d	−0.05	−1.09
3	Empirical	Biesbroek	2017	HIC/MIC	Dietary Guidelines	All-cause Mortality/Morbidity	deaths per 100000PY	20.91	4.40	GHG	kgCO2e/d	0.09	2.04
3	Empirical	Biesbroek	2017	HIC/MIC	Dietary Guidelines	All-cause Mortality/Morbidity	deaths per 100000PY	114.60	19.21	GHG	kgCO2e/d	0.07	1.94
3	Empirical	Biesbroek	2017	HIC/MIC	Dietary Guidelines	All-cause Mortality/Morbidity	deaths per 100000PY	−77.40	−12.98	GHG	kgCO2e/d	−0.10	−2.53
3	Empirical	Biesbroek	2017	HIC/MIC	Dietary Guidelines	All-cause Mortality/Morbidity	deaths per 100000PY	5.73	0.96	GHG	kgCO2e/d	0.06	1.73
3	Empirical	Biesbroek	2017	HIC/MIC	Dietary Guidelines	All-cause Mortality/Morbidity	deaths per 100000PY	138.62	27.54	LU	m2*year/d	0.14	3.09
3	Empirical	Biesbroek	2017	HIC/MIC	Dietary Guidelines	All-cause Mortality/Morbidity	deaths per 100000PY	−12.20	−2.42	LU	m2*year/d	0.03	0.70

(Continued)

(continued).

Study number	Type of study	First author	Year of publication	Region	Final diet category	Health category	Health unit	Health—absolute difference	Health—relative difference	Env outcome	Env unit	Environment—absolute difference	Environment—relative difference
3	Empirical	Biesbroek	2017	HIC/MIC	Dietary Guidelines	All-cause Mortality/Morbidity	deaths per 100000PY	20.91	4.40	LU	m2*year/d	0.12	2.77
3	Empirical	Biesbroek	2017	HIC/MIC	Dietary Guidelines	All-cause Mortality/Morbidity	deaths per 100000PY	114.60	19.21	LU	m2*year/d	0.03	0.81
3	Empirical	Biesbroek	2017	HIC/MIC	Dietary Guidelines	All-cause Mortality/Morbidity	deaths per 100000PY	−77.40	−12.98	LU	m2*year/d	0.00	0.00
3	Empirical	Biesbroek	2017	HIC/MIC	Dietary Guidelines	All-cause Mortality/Morbidity	deaths per 100000PY	5.73	0.96	LU	m2*year/d	0.15	4.38
4	Modelling	Chen	2019	HIC/MIC	Dietary Guidelines	DRCD	DALYs	−953.00	−0.16	GHG	kgCO2e/d	−1.06	−46.98
4	Modelling	Chen	2019	HIC/MIC	Dietary Guidelines	DRCD	DALYs	−15 756.00	−2.67	GHG	kgCO2e/d	−1.23	−54.30
4	Modelling	Chen	2019	HIC/MIC	Dietary Guidelines	CVD	DALYs	419.29	0.33	GHG	kgCO2e/d	−1.06	−46.98
4	Modelling	Chen	2019	HIC/MIC	Dietary Guidelines	CVD	DALYs	−5424.19	−4.33	GHG	kgCO2e/d	−1.23	−54.30
4	Modelling	Chen	2019	HIC/MIC	Dietary Guidelines	CVD	DALYs	531.41	0.90	GHG	kgCO2e/d	−1.06	−46.98
4	Modelling	Chen	2019	HIC/MIC	Dietary Guidelines	CVD	DALYs	−2605.05	−4.41	GHG	kgCO2e/d	−1.23	−54.30
4	Modelling	Chen	2019	HIC/MIC	Dietary Guidelines	DRCD	DALYs	−953.00	−0.16	WU	m3	−0.19	−32.33
4	Modelling	Chen	2019	HIC/MIC	Dietary Guidelines	DRCD	DALYs	−15 756.00	−2.67	WU	m3	−0.15	−26.10
4	Modelling	Chen	2019	HIC/MIC	Vegan	DRCD	DALYs	−20 986.00	−3.55	WU	m3	0.01	2.34
4	Modelling	Chen	2019	HIC/MIC	Vegetarian	DRCD	DALYs	−8049.00	−1.36	WU	m3	0.02	3.51
4	Modelling	Chen	2019	HIC/MIC	Pescatarian/increase fish	DRCD	DALYs	−10 679.00	−1.81	WU	m3	0.02	2.90
4	Modelling	Chen	2019	HIC/MIC	Flexitarian	DRCD	DALYs	−5259.00	−0.89	WU	m3	0.00	0.02
4	Modelling	Chen	2019	HIC/MIC	Dietary Guidelines	DRCD	DALYs	−953.00	−0.16	LU	m2	−1.15	−26.15

(Continued)

(continued).

Study number	Type of study	First author	Year of publication	Region	Final diet category	Health category	Health unit	Health—absolute difference	Health—relative difference	Env outcome	Env unit	Environment—absolute difference	Environment—relative difference
4	Modelling	Chen	2019	HIC/MIC	Dietary Guidelines	DRCD	DALYs	−15756.00	−2.67	LU	m2	−1.42	−32.41
4	Modelling	Chen	2019	HIC/MIC	Vegan	DRCD	DALYs	−20986.00	−3.55	LU	m2	−0.31	−6.97
4	Modelling	Chen	2019	HIC/MIC	Vegetarian	DRCD	DALYs	−8049.00	−1.36	LU	m2	−0.12	−2.63
4	Modelling	Chen	2019	HIC/MIC	Pescatarian/ increase fish	DRCD	DALYs	−10679.00	−1.81	LU	m2	−0.17	−3.87
4	Modelling	Chen	2019	HIC/MIC	Flexitarian	DRCD	DALYs	−5259.00	−0.89	LU	m2	−0.22	−4.96
4	Modelling	Chen	2019	HIC/MIC	Dietary Guidelines	DRCD	DALYs	−953.00	−0.16	NU	g/d	−8.03	−27.67
4	Modelling	Chen	2019	HIC/MIC	Dietary Guidelines	DRCD	DALYs	−15756.00	−2.67	NU	g/d	−9.72	−33.48
4	Modelling	Chen	2019	HIC/MIC	Vegan	DRCD	DALYs	−20986.00	−3.55	NU	g/d	−5.47	−18.83
4	Modelling	Chen	2019	HIC/MIC	Vegetarian	DRCD	DALYs	−8049.00	−1.36	NU	g/d	−3.36	−11.57
4	Modelling	Chen	2019	HIC/MIC	Pescatarian/ increase fish	DRCD	DALYs	−10679.00	−1.81	NU	g/d	−3.33	−11.46
4	Modelling	Chen	2019	HIC/MIC	Flexitarian	DRCD	DALYs	−5259.00	−0.89	NU	g/d	−3.00	−10.35
4	Modelling	Chen	2019	HIC/MIC	Dietary Guidelines	DRCD	DALYs	−953.00	−0.16	PU	g/d	−1.49	−28.40
4	Modelling	Chen	2019	HIC/MIC	Dietary Guidelines	DRCD	DALYs	−15756.00	−2.67	PU	g/d	−1.79	−34.12
4	Modelling	Chen	2019	HIC/MIC	Vegan	DRCD	DALYs	−20986.00	−3.55	PU	g/d	−0.90	−17.29
4	Modelling	Chen	2019	HIC/MIC	Vegetarian	DRCD	DALYs	−8049.00	−1.36	PU	g/d	−0.62	−11.91
4	Modelling	Chen	2019	HIC/MIC	Pescatarian/ increase fish	DRCD	DALYs	−10679.00	−1.81	PU	g/d	−0.61	−11.63

(Continued)

(continued).

Study number	Type of study	First author	Year of publication	Region	Final diet category	Health category	Health unit	Health—absolute difference	Health—relative difference	Env outcome	Env unit	Environment—absolute difference	Environment—relative difference
4	Modelling	Chen	2019	HIC/MIC	Flexitarian	DRCD	DALYs	−5259.00	−0.89	PU	g/d	−0.55	−10.42
4	Modelling	Chen	2019	HIC/MIC	Dietary Guidelines	Diabetes	DALYs	−2341.99	−3.41	GHG	kgCO2e/d	−1.06	−46.98
4	Modelling	Chen	2019	HIC/MIC	Dietary Guidelines	Diabetes	DALYs	−2617.28	−3.81	GHG	kgCO2e/d	−1.23	−54.30
4	Modelling	Chen	2019	HIC/MIC	Dietary Guidelines	Cancer	DALYs	438.58	0.12	GHG	kgCO2e/d	−1.06	−46.98
4	Modelling	Chen	2019	HIC/MIC	Dietary Guidelines	Cancer	DALYs	−5109.90	−1.43	GHG	kgCO2e/d	−1.23	−54.30
4	Modelling	Chen	2019	HIC/MIC	Flexitarian	DRCD	DALYs	−5259.00	−0.89	GHG	kgCO2e/d	−1.03	−45.37
4	Modelling	Chen	2019	HIC/MIC	Flexitarian	CVD	DALYs	−1369.39	−1.09	GHG	kgCO2e/d	−1.03	−45.37
4	Modelling	Chen	2019	HIC/MIC	Dietary Guidelines	CVD	DALYs	419.29	0.33	WU	m3	−0.19	−32.33
4	Modelling	Chen	2019	HIC/MIC	Dietary Guidelines	CVD	DALYs	−5424.19	−4.33	WU	m3	−0.15	−26.10
4	Modelling	Chen	2019	HIC/MIC	Vegan	CVD	DALYs	−9785.20	−7.82	WU	m3	0.01	2.34
4	Modelling	Chen	2019	HIC/MIC	Vegetarian	CVD	DALYs	−1967.18	−1.57	WU	m3	0.02	3.51
4	Modelling	Chen	2019	HIC/MIC	Pescatarian/ increase fish	CVD	DALYs	−4473.01	−3.57	WU	m3	0.02	2.90
4	Modelling	Chen	2019	HIC/MIC	Flexitarian	CVD	DALYs	−1369.39	−1.09	WU	m3	0.00	0.02
4	Modelling	Chen	2019	HIC/MIC	Dietary Guidelines	CVD	DALYs	419.29	0.33	LU	m2	−1.15	−26.15
4	Modelling	Chen	2019	HIC/MIC	Dietary Guidelines	CVD	DALYs	−5424.19	−4.33	LU	m2	−1.42	−32.41
4	Modelling	Chen	2019	HIC/MIC	Vegan	CVD	DALYs	−9785.20	−7.82	LU	m2	−0.31	−6.97
4	Modelling	Chen	2019	HIC/MIC	Vegetarian	CVD	DALYs	−1967.18	−1.57	LU	m2	−0.12	−2.63
4	Modelling	Chen	2019	HIC/MIC	Pescatarian/ increase fish	CVD	DALYs	−4473.01	−3.57	LU	m2	−0.17	−3.87

(Continued)

(continued).

Study number	Type of study	First author	Year of publication	Region	Final diet category	Health category	Health unit	Health—absolute difference	Health—relative difference	Env outcome	Env unit	Environment—absolute difference	Environment—relative difference
4	Modelling	Chen	2019	HIC/MIC	Flexitarian	CVD	DALYs	−1369.39	−1.09	LU	m2	−0.22	−4.96
4	Modelling	Chen	2019	HIC/MIC	Dietary Guidelines	CVD	DALYs	419.29	0.33	NU	g/d	−8.03	−27.67
4	Modelling	Chen	2019	HIC/MIC	Dietary Guidelines	CVD	DALYs	−5424.19	−4.33	NU	g/d	−9.72	−33.48
4	Modelling	Chen	2019	HIC/MIC	Vegan	CVD	DALYs	−9785.20	−7.82	NU	g/d	−5.47	−18.83
4	Modelling	Chen	2019	HIC/MIC	Vegetarian	CVD	DALYs	−1967.18	−1.57	NU	g/d	−3.36	−11.57
4	Modelling	Chen	2019	HIC/MIC	Pescatarian/ increase fish	CVD	DALYs	−4473.01	−3.57	NU	g/d	−3.33	−11.46
4	Modelling	Chen	2019	HIC/MIC	Flexitarian	CVD	DALYs	−1369.39	−1.09	NU	g/d	−3.00	−10.35
4	Modelling	Chen	2019	HIC/MIC	Dietary Guidelines	CVD	DALYs	419.29	0.33	PU	g/d	−1.49	−28.40
4	Modelling	Chen	2019	HIC/MIC	Dietary Guidelines	CVD	DALYs	−5424.19	−4.33	PU	g/d	−1.79	−34.12
4	Modelling	Chen	2019	HIC/MIC	Vegan	CVD	DALYs	−9785.20	−7.82	PU	g/d	−0.90	−17.29
4	Modelling	Chen	2019	HIC/MIC	Vegetarian	CVD	DALYs	−1967.18	−1.57	PU	g/d	−0.62	−11.91
4	Modelling	Chen	2019	HIC/MIC	Pescatarian/ increase fish	CVD	DALYs	−4473.01	−3.57	PU	g/d	−0.61	−11.63
4	Modelling	Chen	2019	HIC/MIC	Flexitarian	CVD	DALYs	−1369.39	−1.09	PU	g/d	−0.55	−10.42
4	Modelling	Chen	2019	HIC/MIC	Flexitarian	CVD	DALYs	−1417.66	−2.40	GHG	kgCO2e/d	−1.03	−45.37
4	Modelling	Chen	2019	HIC/MIC	Flexitarian	Diabetes	DALYs	−625.75	−0.91	GHG	kgCO2e/d	−1.03	−45.37
4	Modelling	Chen	2019	HIC/MIC	Flexitarian	Cancer	DALYs	−1846.29	−0.52	GHG	kgCO2e/d	−1.03	−45.37
4	Modelling	Chen	2019	HIC/MIC	Pescatarian/ increase fish	DRCD	DALYs	−10 679.00	−1.81	GHG	kgCO2e/d	−1.49	−65.65
4	Modelling	Chen	2019	HIC/MIC	Pescatarian/ increase fish	CVD	DALYs	−4473.01	−3.57	GHG	kgCO2e/d	−1.49	−65.65
4	Modelling	Chen	2019	HIC/MIC	Pescatarian/ increase fish	CVD	DALYs	−2394.50	−4.06	GHG	kgCO2e/d	−1.49	−65.65

(Continued)

(continued).

Study number	Type of study	First author	Year of publication	Region	Final diet category	Health category	Health unit	Health—absolute difference	Health—relative difference	Env outcome	Env unit	Environment—absolute difference	Environment—relative difference
4	Modelling	Chen	2019	HIC/MIC	Dietary Guidelines	CVD	DALYs	531.41	0.90	WU	m3	−0.19	−32.33
4	Modelling	Chen	2019	HIC/MIC	Dietary Guidelines	CVD	DALYs	−2605.05	−4.41	WU	m3	−0.15	−26.10
4	Modelling	Chen	2019	HIC/MIC	Vegan	CVD	DALYs	−3033.43	−5.14	WU	m3	0.01	2.34
4	Modelling	Chen	2019	HIC/MIC	Vegetarian	CVD	DALYs	−2382.34	−4.03	WU	m3	0.02	3.51
4	Modelling	Chen	2019	HIC/MIC	Pescatarian/ increase fish	CVD	DALYs	−2394.50	−4.06	WU	m3	0.02	2.90
4	Modelling	Chen	2019	HIC/MIC	Flexitarian	CVD	DALYs	−1417.66	−2.40	WU	m3	0.00	0.02
4	Modelling	Chen	2019	HIC/MIC	Dietary Guidelines	CVD	DALYs	531.41	0.90	LU	m2	−1.15	−26.15
4	Modelling	Chen	2019	HIC/MIC	Dietary Guidelines	CVD	DALYs	−2605.05	−4.41	LU	m2	−1.42	−32.41
4	Modelling	Chen	2019	HIC/MIC	Vegan	CVD	DALYs	−3033.43	−5.14	LU	m2	−0.31	−6.97
4	Modelling	Chen	2019	HIC/MIC	Vegetarian	CVD	DALYs	−2382.34	−4.03	LU	m2	−0.12	−2.63
4	Modelling	Chen	2019	HIC/MIC	Pescatarian/ increase fish	CVD	DALYs	−2394.50	−4.06	LU	m2	−0.17	−3.87
4	Modelling	Chen	2019	HIC/MIC	Flexitarian	CVD	DALYs	−1417.66	−2.40	LU	m2	−0.22	−4.96
4	Modelling	Chen	2019	HIC/MIC	Dietary Guidelines	CVD	DALYs	531.41	0.90	NU	g/d	−8.03	−27.67
4	Modelling	Chen	2019	HIC/MIC	Dietary Guidelines	CVD	DALYs	−2605.05	−4.41	NU	g/d	−9.72	−33.48
4	Modelling	Chen	2019	HIC/MIC	Vegan	CVD	DALYs	−3033.43	−5.14	NU	g/d	−5.47	−18.83
4	Modelling	Chen	2019	HIC/MIC	Vegetarian	CVD	DALYs	−2382.34	−4.03	NU	g/d	−3.36	−11.57
4	Modelling	Chen	2019	HIC/MIC	Pescatarian/ increase fish	CVD	DALYs	−2394.50	−4.06	NU	g/d	−3.33	−11.46
4	Modelling	Chen	2019	HIC/MIC	Flexitarian	CVD	DALYs	−1417.66	−2.40	NU	g/d	−3.00	−10.35
4	Modelling	Chen	2019	HIC/MIC	Dietary Guidelines	CVD	DALYs	531.41	0.90	PU	g/d	−1.49	−28.40
4	Modelling	Chen	2019	HIC/MIC	Dietary Guidelines	CVD	DALYs	−2605.05	−4.41	PU	g/d	−1.79	−34.12

(Continued)

(continued).

Study number	Type of study	First author	Year of publication	Region	Final diet category	Health category	Health unit	Health—absolute difference	Health—relative difference	Env outcome	Env unit	Environment—absolute difference	Environment—relative difference
4	Modelling	Chen	2019	HIC/MIC	Vegan	CVD	DALYs	−3033.43	−5.14	PU	g/d	−0.90	−17.29
4	Modelling	Chen	2019	HIC/MIC	Vegetarian	CVD	DALYs	−2382.34	−4.03	PU	g/d	−0.62	−11.91
4	Modelling	Chen	2019	HIC/MIC	Pescatarian/ increase fish	CVD	DALYs	−2394.50	−4.06	PU	g/d	−0.61	−11.63
4	Modelling	Chen	2019	HIC/MIC	Flexitarian	CVD	DALYs	−1417.66	−2.40	PU	g/d	−0.55	−10.42
4	Modelling	Chen	2019	HIC/MIC	Pescatarian/ increase fish	Diabetes	DALYs	−605.54	−0.88	GHG	kgCO2e/d	−1.49	−65.65
4	Modelling	Chen	2019	HIC/MIC	Pescatarian/ increase fish	Cancer	DALYs	−3206.16	−0.90	GHG	kgCO2e/d	−1.49	−65.65
4	Modelling	Chen	2019	HIC/MIC	Vegan	DRCD	DALYs	−20 986.00	−3.55	GHG	kgCO2e/d	−1.89	−83.39
4	Modelling	Chen	2019	HIC/MIC	Vegan	CVD	DALYs	−9785.20	−7.82	GHG	kgCO2e/d	−1.89	−83.39
4	Modelling	Chen	2019	HIC/MIC	Vegan	CVD	DALYs	−3033.43	−5.14	GHG	kgCO2e/d	−1.89	−83.39
4	Modelling	Chen	2019	HIC/MIC	Vegan	Diabetes	DALYs	−1150.42	−1.67	GHG	kgCO2e/d	−1.89	−83.39
4	Modelling	Chen	2019	HIC/MIC	Dietary Guidelines	Diabetes	DALYs	−2341.99	−3.41	WU	m3	−0.19	−32.33
4	Modelling	Chen	2019	HIC/MIC	Dietary Guidelines	Diabetes	DALYs	−2617.28	−3.81	WU	m3	−0.15	−26.10
4	Modelling	Chen	2019	HIC/MIC	Vegan	Diabetes	DALYs	−1150.42	−1.67	WU	m3	0.01	2.34
4	Modelling	Chen	2019	HIC/MIC	Vegetarian	Diabetes	DALYs	−550.66	−0.80	WU	m3	0.02	3.51
4	Modelling	Chen	2019	HIC/MIC	Pescatarian/ increase fish	Diabetes	DALYs	−605.54	−0.88	WU	m3	0.02	2.90
4	Modelling	Chen	2019	HIC/MIC	Flexitarian	Diabetes	DALYs	−625.75	−0.91	WU	m3	0.00	0.02
4	Modelling	Chen	2019	HIC/MIC	Dietary Guidelines	Diabetes	DALYs	−2341.99	−3.41	LU	m2	−1.15	−26.15
4	Modelling	Chen	2019	HIC/MIC	Dietary Guidelines	Diabetes	DALYs	−2617.28	−3.81	LU	m2	−1.42	−32.41
4	Modelling	Chen	2019	HIC/MIC	Vegan	Diabetes	DALYs	−1150.42	−1.67	LU	m2	−0.31	−6.97
4	Modelling	Chen	2019	HIC/MIC	Vegetarian	Diabetes	DALYs	−550.66	−0.80	LU	m2	−0.12	−2.63
4	Modelling	Chen	2019	HIC/MIC	Pescatarian/ increase fish	Diabetes	DALYs	−605.54	−0.88	LU	m2	−0.17	−3.87
4	Modelling	Chen	2019	HIC/MIC	Flexitarian	Diabetes	DALYs	−625.75	−0.91	LU	m2	−0.22	−4.96

(Continued)

(continued).

Study number	Type of study	First author	Year of publication	Region	Final diet category	Health category	Health unit	Health—absolute difference	Health—relative difference	Env outcome	Env unit	Environment—absolute difference	Environment—relative difference
4	Modelling	Chen	2019	HIC/MIC	Dietary Guidelines	Diabetes	DALYs	−2341.99	−3.41	NU	g/d	−8.03	−27.67
4	Modelling	Chen	2019	HIC/MIC	Dietary Guidelines	Diabetes	DALYs	−2617.28	−3.81	NU	g/d	−9.72	−33.48
4	Modelling	Chen	2019	HIC/MIC	Vegan	Diabetes	DALYs	−1150.42	−1.67	NU	g/d	−5.47	−18.83
4	Modelling	Chen	2019	HIC/MIC	Vegetarian	Diabetes	DALYs	−550.66	−0.80	NU	g/d	−3.36	−11.57
4	Modelling	Chen	2019	HIC/MIC	Pescatarian/ increase fish	Diabetes	DALYs	−605.54	−0.88	NU	g/d	−3.33	−11.46
4	Modelling	Chen	2019	HIC/MIC	Flexitarian	Diabetes	DALYs	−625.75	−0.91	NU	g/d	−3.00	−10.35
4	Modelling	Chen	2019	HIC/MIC	Dietary Guidelines	Diabetes	DALYs	−2341.99	−3.41	PU	g/d	−1.49	−28.40
4	Modelling	Chen	2019	HIC/MIC	Dietary Guidelines	Diabetes	DALYs	−2617.28	−3.81	PU	g/d	−1.79	−34.12
4	Modelling	Chen	2019	HIC/MIC	Vegan	Diabetes	DALYs	−1150.42	−1.67	PU	g/d	−0.90	−17.29
4	Modelling	Chen	2019	HIC/MIC	Vegetarian	Diabetes	DALYs	−550.66	−0.80	PU	g/d	−0.62	−11.91
4	Modelling	Chen	2019	HIC/MIC	Pescatarian/ increase fish	Diabetes	DALYs	−605.54	−0.88	PU	g/d	−0.61	−11.63
4	Modelling	Chen	2019	HIC/MIC	Flexitarian	Diabetes	DALYs	−625.75	−0.91	PU	g/d	−0.55	−10.42
4	Modelling	Chen	2019	HIC/MIC	Vegan	Cancer	DALYs	−7016.77	−1.96	GHG	kgCO2e/d	−1.89	−83.39
4	Modelling	Chen	2019	HIC/MIC	Vegetarian	DRCD	DALYs	−8049.00	−1.36	GHG	kgCO2e/d	−1.48	−65.46
4	Modelling	Chen	2019	HIC/MIC	Vegetarian	CVD	DALYs	−1967.18	−1.57	GHG	kgCO2e/d	−1.48	−65.46
4	Modelling	Chen	2019	HIC/MIC	Vegetarian	CVD	DALYs	−2382.34	−4.03	GHG	kgCO2e/d	−1.48	−65.46
4	Modelling	Chen	2019	HIC/MIC	Vegetarian	Diabetes	DALYs	−550.66	−0.80	GHG	kgCO2e/d	−1.48	−65.46
4	Modelling	Chen	2019	HIC/MIC	Vegetarian	Cancer	DALYs	−3148.82	−0.88	GHG	kgCO2e/d	−1.48	−65.46
4	Modelling	Chen	2019	HIC/MIC	Dietary Guidelines	Cancer	DALYs	438.58	0.12	WU	m3	−0.19	−32.33

(Continued)

(continued).

Study number	Type of study	First author	Year of publication	Region	Final diet category	Health category	Health unit	Health—absolute difference	Health—relative difference	Env outcome	Env unit	Environment—absolute difference	Environment—relative difference
4	Modelling	Chen	2019	HIC/MIC	Dietary Guidelines	Cancer	DALYs	−5109.90	−1.43	WU	m3	−0.15	−26.10
4	Modelling	Chen	2019	HIC/MIC	Vegan	Cancer	DALYs	−7016.77	−1.96	WU	m3	0.01	2.34
4	Modelling	Chen	2019	HIC/MIC	Vegetarian	Cancer	DALYs	−3148.82	−0.88	WU	m3	0.02	3.51
4	Modelling	Chen	2019	HIC/MIC	Pescatarian/ increase fish	Cancer	DALYs	−3206.16	−0.90	WU	m3	0.02	2.90
4	Modelling	Chen	2019	HIC/MIC	Flexitarian	Cancer	DALYs	−1846.29	−0.52	WU	m3	0.00	0.02
4	Modelling	Chen	2019	HIC/MIC	Dietary Guidelines	Cancer	DALYs	438.58	0.12	LU	m2	−1.15	−26.15
4	Modelling	Chen	2019	HIC/MIC	Dietary Guidelines	Cancer	DALYs	−5109.90	−1.43	LU	m2	−1.42	−32.41
4	Modelling	Chen	2019	HIC/MIC	Vegan	Cancer	DALYs	−7016.77	−1.96	LU	m2	−0.31	−6.97
4	Modelling	Chen	2019	HIC/MIC	Vegetarian	Cancer	DALYs	−3148.82	−0.88	LU	m2	−0.12	−2.63
4	Modelling	Chen	2019	HIC/MIC	Pescatarian/ increase fish	Cancer	DALYs	−3206.16	−0.90	LU	m2	−0.17	−3.87
4	Modelling	Chen	2019	HIC/MIC	Flexitarian	Cancer	DALYs	−1846.29	−0.52	LU	m2	−0.22	−4.96
4	Modelling	Chen	2019	HIC/MIC	Dietary Guidelines	Cancer	DALYs	438.58	0.12	NU	g/d	−8.03	−27.67
4	Modelling	Chen	2019	HIC/MIC	Dietary Guidelines	Cancer	DALYs	−5109.90	−1.43	NU	g/d	−9.72	−33.48
4	Modelling	Chen	2019	HIC/MIC	Vegan	Cancer	DALYs	−7016.77	−1.96	NU	g/d	−5.47	−18.83
4	Modelling	Chen	2019	HIC/MIC	Vegetarian	Cancer	DALYs	−3148.82	−0.88	NU	g/d	−3.36	−11.57
4	Modelling	Chen	2019	HIC/MIC	Pescatarian/ increase fish	Cancer	DALYs	−3206.16	−0.90	NU	g/d	−3.33	−11.46
4	Modelling	Chen	2019	HIC/MIC	Flexitarian	Cancer	DALYs	−1846.29	−0.52	NU	g/d	−3.00	−10.35
4	Modelling	Chen	2019	HIC/MIC	Dietary Guidelines	Cancer	DALYs	438.58	0.12	PU	g/d	−1.49	−28.40
4	Modelling	Chen	2019	HIC/MIC	Dietary Guidelines	Cancer	DALYs	−5109.90	−1.43	PU	g/d	−1.79	−34.12
4	Modelling	Chen	2019	HIC/MIC	Vegan	Cancer	DALYs	−7016.77	−1.96	PU	g/d	−0.90	−17.29
4	Modelling	Chen	2019	HIC/MIC	Vegetarian	Cancer	DALYs	−3148.82	−0.88	PU	g/d	−0.62	−11.91
4	Modelling	Chen	2019	HIC/MIC	Pescatarian/ increase fish	Cancer	DALYs	−3206.16	−0.90	PU	g/d	−0.61	−11.63

(Continued)

(continued).

Study number	Type of study	First author	Year of publication	Region	Final diet category	Health category	Health unit	Health—absolute difference	Health—relative difference	Env outcome	Env unit	Environment—absolute difference	Environment—relative difference
4	Modelling	Chen	2019	HIC/MIC	Flexitarian	Cancer	DALYs	−1846.29	−0.52	PU	g/d	−0.55	−10.42
5	Modelling	Cobiac	2019	HIC/MIC	Substitute ASF with PSF	All-cause Mortality/Morbidity	DALYs	−28 000 000.00	−11.35	GHG	%reduction		0.00
5	Modelling	Cobiac	2019	HIC/MIC	Substitute ASF with PSF	All-cause Mortality/Morbidity	DALYs	−28 000 000.00	−11.35	GHG	%reduction		−10.00
5	Modelling	Cobiac	2019	HIC/MIC	Substitute ASF with PSF	All-cause Mortality/Morbidity	DALYs	−28 000 000.00	−11.35	GHG	%reduction		−20.00
5	Modelling	Cobiac	2019	HIC/MIC	Substitute ASF with PSF	All-cause Mortality/Morbidity	DALYs	−31 000 000.00	−12.57	GHG	%reduction		−30.00
5	Modelling	Cobiac	2019	HIC/MIC	Substitute ASF with PSF	All-cause Mortality/Morbidity	DALYs	−33 000 000.00	−13.38	GHG	%reduction		−40.00
5	Modelling	Cobiac	2019	HIC/MIC	Substitute ASF with PSF	All-cause Mortality/Morbidity	DALYs	−39 000 000.00	−15.81	GHG	%reduction		−50.00
5	Modelling	Cobiac	2019	HIC/MIC	Substitute ASF with PSF	All-cause Mortality/Morbidity	DALYs	−39 000 000.00	−15.81	GHG	%reduction		−60.00
5	Modelling	Cobiac	2019	HIC/MIC	Substitute ASF with PSF	All-cause Mortality/Morbidity	DALYs	−14 500 000.00	−6.23	GHG	%reduction		0.00
5	Modelling	Cobiac	2019	HIC/MIC	Substitute ASF with PSF	All-cause Mortality/Morbidity	DALYs	−13 200 000.00	−5.67	GHG	%reduction		−10.00
5	Modelling	Cobiac	2019	HIC/MIC	Substitute ASF with PSF	All-cause Mortality/Morbidity	DALYs	−13 200 000.00	−5.67	GHG	%reduction		−20.00
5	Modelling	Cobiac	2019	HIC/MIC	Substitute ASF with PSF	All-cause Mortality/Morbidity	DALYs	−13 600 000.00	−5.84	GHG	%reduction		−30.00
5	Modelling	Cobiac	2019	HIC/MIC	Substitute ASF with PSF	All-cause Mortality/Morbidity	DALYs	−12 300 000.00	−5.29	GHG	%reduction		−40.00
5	Modelling	Cobiac	2019	HIC/MIC	Substitute ASF with PSF	All-cause Mortality/Morbidity	DALYs	−10 500 000.00	−4.51	GHG	%reduction		−50.00
5	Modelling	Cobiac	2019	HIC/MIC	Substitute ASF with PSF	All-cause Mortality/Morbidity	DALYs	−17 200 000.00	−7.39	GHG	%reduction		−60.00
5	Modelling	Cobiac	2019	HIC/MIC	Substitute ASF with PSF	All-cause Mortality/Morbidity	DALYs	−9 800 000.00	−4.24	GHG	%reduction		0.00

(Continued)

(continued).

Study number	Type of study	First author	Year of publication	Region	Final diet category	Health category	Health unit	Health—absolute difference	Health—relative difference	Env outcome	Env unit	Environment—absolute difference	Environment—relative difference
5	Modelling	Cobiac	2019	HIC/MIC	Substitute ASF with PSF	All-cause Mortality/Morbidity	DALYs	−9 500 000.00	−4.11	GHG	%reduction		−10.00
5	Modelling	Cobiac	2019	HIC/MIC	Substitute ASF with PSF	All-cause Mortality/Morbidity	DALYs	−9 000 000.00	−3.89	GHG	%reduction		−20.00
5	Modelling	Cobiac	2019	HIC/MIC	Substitute ASF with PSF	All-cause Mortality/Morbidity	DALYs	−10 000 000.00	−4.33	GHG	%reduction		−30.00
5	Modelling	Cobiac	2019	HIC/MIC	Substitute ASF with PSF	All-cause Mortality/Morbidity	DALYs	−10 400 000.00	−4.50	GHG	%reduction		−40.00
5	Modelling	Cobiac	2019	HIC/MIC	Substitute ASF with PSF	All-cause Mortality/Morbidity	DALYs	−18 700 000.00	−8.09	GHG	%reduction		−50.00
5	Modelling	Cobiac	2019	HIC/MIC	Substitute ASF with PSF	All-cause Mortality/Morbidity	DALYs	−27 100 000.00	−11.72	GHG	%reduction		−60.00
5	Modelling	Cobiac	2019	HIC/MIC	Substitute ASF with PSF	All-cause Mortality/Morbidity	DALYs	−2 100 000.00	−5.87	GHG	%reduction		0.00
5	Modelling	Cobiac	2019	HIC/MIC	Substitute ASF with PSF	All-cause Mortality/Morbidity	DALYs	−2 100 000.00	−5.87	GHG	%reduction		−10.00
5	Modelling	Cobiac	2019	HIC/MIC	Substitute ASF with PSF	All-cause Mortality/Morbidity	DALYs	−2 000 000.00	−5.59	GHG	%reduction		−20.00
5	Modelling	Cobiac	2019	HIC/MIC	Substitute ASF with PSF	All-cause Mortality/Morbidity	DALYs	−2 200 000.00	−6.15	GHG	%reduction		−30.00
5	Modelling	Cobiac	2019	HIC/MIC	Substitute ASF with PSF	All-cause Mortality/Morbidity	DALYs	−2 800 000.00	−7.83	GHG	%reduction		−40.00
5	Modelling	Cobiac	2019	HIC/MIC	Substitute ASF with PSF	All-cause Mortality/Morbidity	DALYs	−2 600 000.00	−7.27	GHG	%reduction		−50.00
5	Modelling	Cobiac	2019	HIC/MIC	Substitute ASF with PSF	All-cause Mortality/Morbidity	DALYs	−3 400 000.00	−9.51	GHG	%reduction		−60.00
5	Modelling	Cobiac	2019	HIC/MIC	Substitute ASF with PSF	All-cause Mortality/Morbidity	DALYs	−3 800 000.00	−16.87	GHG	%reduction		0.00

(Continued)

(continued).

Study number	Type of study	First author	Year of publication	Region	Final diet category	Health category	Health unit	Health—absolute difference	Health—relative difference	Env outcome	Env unit	Environment—absolute difference	Environment—relative difference
5	Modelling	Cobiac	2019	HIC/MIC	Substitute ASF with PSF	All-cause Mortality/ Morbidity	DALYs	−4 000 000.00	−17.75	GHG	%reduction		−10.00
5	Modelling	Cobiac	2019	HIC/MIC	Substitute ASF with PSF	All-cause Mortality/ Morbidity	DALYs	−4 000 000.00	−17.75	GHG	%reduction		−20.00
5	Modelling	Cobiac	2019	HIC/MIC	Substitute ASF with PSF	All-cause Mortality/ Morbidity	DALYs	−4 000 000.00	−17.75	GHG	%reduction		−30.00
5	Modelling	Cobiac	2019	HIC/MIC	Substitute ASF with PSF	All-cause Mortality/ Morbidity	DALYs	−4 400 000.00	−19.53	GHG	%reduction		−40.00
5	Modelling	Cobiac	2019	HIC/MIC	Substitute ASF with PSF	All-cause Mortality/ Morbidity	DALYs	−4 300 000.00	−19.09	GHG	%reduction		−50.00
5	Modelling	Cobiac	2019	HIC/MIC	Substitute ASF with PSF	All-cause Mortality/ Morbidity	DALYs	−4 500 000.00	−19.97	GHG	%reduction		−60.00
5	Modelling	Cobiac	2019	HIC/MIC	Substitute ASF with PSF	All-cause Mortality/ Morbidity	DALYs	−4 700 000.00	−20.86	GHG	%reduction		−70.00
6	Modelling	Farchi	2017	HIC/MIC	Mediterranean	Cancer	% Deaths Avoided	−3.70	−3.70	GHG	Annual GHG Emis-sions Italy in Gg CO2 eqv	−8160.00	−63.06
6	Modelling	Farchi	2017	HIC/MIC	Mediterranean	CVD	% Deaths Avoided	−3.30	−3.30	GHG	Annual GHG Emis-sions Italy in Gg CO2 eqv	−8160.00	−63.06
7	Empirical	Fresan	2018	HIC/MIC	Mediterranean	All-cause Mortality/ Morbidity	Incidence Rate (%)	−0.10	−6.88	Other	EFI	−0.11	−1.10

(Continued)

(continued).

Study number	Type of study	First author	Year of publication	Region	Final diet category	Health category	Health unit	Health—absolute difference	Health—relative difference	Env outcome	Env unit	Environment—absolute difference	Environment—relative difference
7	Empirical	Fresan	2018	HIC/MIC	Mediterranean	Cancer	Incidence Rate (%)	0.12	35.45	Other	EFI	−0.11	−1.10
7	Empirical	Fresan	2018	HIC/MIC	Mediterranean	CVD	Incidence Rate (%)	0.11	17.58	Other	EFI	−0.11	−1.10
7	Empirical	Fresan	2018	HIC/MIC	Mediterranean	Diabetes	Incidence Rate (%)	0.07	10.23	Other	EFI	−0.11	−1.10
7	Empirical	Fresan	2018	HIC/MIC	Vegetarian	All-cause Mortality/Morbidity	Incidence Rate (%)	−0.04	−3.15	Other	EFI	−0.28	−2.82
7	Empirical	Fresan	2018	HIC/MIC	Vegetarian	Cancer	Incidence Rate (%)	0.03	13.70	Other	EFI	−0.28	−2.82
7	Empirical	Fresan	2018	HIC/MIC	Vegetarian	CVD	Incidence Rate (%)	0.02	3.53	Other	EFI	−0.28	−2.82
7	Empirical	Fresan	2018	HIC/MIC	Vegetarian	Diabetes	Incidence Rate (%)	−0.04	−5.73	Other	EFI	−0.28	−2.82
7	Empirical	Fresan	2018	HIC/MIC	Mediterranean	All-cause Mortality/Morbidity	Incidence Rate (%)	−0.15	−10.68	Other	EFI	−0.53	−5.29
7	Empirical	Fresan	2018	HIC/MIC	Mediterranean	Cancer	Incidence Rate (%)	−0.10	−28.54	Other	EFI	−0.53	−5.29
7	Empirical	Fresan	2018	HIC/MIC	Mediterranean	CVD	Incidence Rate (%)	−0.11	−16.99	Other	EFI	−0.53	−5.29
7	Empirical	Fresan	2018	HIC/MIC	Mediterranean	Diabetes	Incidence Rate (%)	0.26	38.72	Other	EFI	−0.53	−5.29
7	Empirical	Fresan	2018	HIC/MIC	Vegetarian	All-cause Mortality/Morbidity	Incidence Rate (%)	0.37	32.15	Other	EFI	−1.11	−11.18
7	Empirical	Fresan	2018	HIC/MIC	Vegetarian	Cancer	Incidence Rate (%)	0.19	78.64	Other	EFI	−1.11	−11.18
7	Empirical	Fresan	2018	HIC/MIC	Vegetarian	CVD	Incidence Rate (%)	−0.11	−16.29	Other	EFI	−1.11	−11.18
7	Empirical	Fresan	2018	HIC/MIC	Vegetarian	Diabetes	Incidence Rate (%)	0.30	49.14	Other	EFI	−1.11	−11.18

(Continued)

(continued).

Study number	Type of study	First author	Year of publication	Region	Final diet category	Health category	Health unit	Health—absolute difference	Health—relative difference	Env outcome	Env unit	Environment—absolute difference	Environment—relative difference
8	Empirical	Hobbs	2019	HIC/MIC	Reduce ASF no substitute	BMI	kg/m^1	0.50	1.82	GHG	kg CO2 eqv/day	−0.05	−1.23
8	Empirical	Hobbs	2019	HIC/MIC	Reduce ASF no substitute	BMI	kg/m^1	0.50	1.82	NU	gN eqv/day	−1.40	−2.68
8	Empirical	Hobbs	2019	HIC/MIC	Reduce ASF no substitute	BMI	kg/m^1	0.50	1.82	Other	g SO2 eqv	1.60	4.66
8	Empirical	Hobbs	2019	HIC/MIC	Reduce ASF no substitute	BMI	kg/m^1	0.50	1.82	GHG	kg CO2 eqv/day	−0.05	−1.23
8	Empirical	Hobbs	2019	HIC/MIC	Reduce ASF no substitute	BMI	kg/m^1	0.50	1.82	NU	gN eqv/day	−1.40	−2.68
8	Empirical	Hobbs	2019	HIC/MIC	Reduce ASF no substitute	BMI	kg/m^1	0.50	1.82	Other	g SO2 eqv	1.60	4.66
9	Modelling	Irz	2016	HIC/MIC	'Sustainable Diet'	DRCD	# Deaths Avoided	2331.00	−3.50	GHG	Change in CO2 emissions (kt)	−2985.00	−5.30
9	Modelling	Irz	2016	HIC/MIC	Reduce ASF no substitute	DRCD	# Deaths Avoided	230.00	−0.30	GHG	Change in CO2 emissions (kt)	−274.00	−0.50
9	Modelling	Irz	2016	HIC/MIC	Reduce ASF no substitute	DRCD	# Deaths Avoided	245.00	−0.40	GHG	Change in CO2 emissions (kt)	−513.00	−0.90
9	Modelling	Irz	2016	HIC/MIC	Substitute ASF with PSF	DRCD	# Deaths Avoided	2507.00	−3.80	GHG	Change in CO2 emissions (kt)	−1574.00	−2.80
9	Modelling	Irz	2016	HIC/MIC	Reduce ASF no substitute	DRCD	# Deaths Avoided	230.00	−0.30	Other	Change in SO2 (kt)	−7.00	−1.00

(Continued)

(continued).

Study number	Type of study	First author	Year of publication	Region	Final diet category	Health category	Health unit	Health—absolute difference	Health—relative difference	Env outcome	Env unit	Environment—absolute difference	Environment—relative difference
10	Modelling	Irz	2017	HIC/MIC	Pescatarian/ increase fish	DRCD	% Deaths Avoided	0.60	−0.60	GHG	% CO2 emissions Avoided		−0.40
9	Modelling	Irz	2016	HIC/MIC	Reduce ASF no substitute	DRCD	# Deaths Avoided	245.00	−0.40	Other	Change in SO2 (kt)	−17.00	−2.50
10	Modelling	Irz	2017	HIC/MIC	Reduce ASF no substitute	DRCD	% Deaths Avoided		−0.30	GHG	% CO2 emissions Avoided		−0.50
9	Modelling	Irz	2016	HIC/MIC	Substitute ASF with PSF	DRCD	# Deaths Avoided	2507.00	−3.80	Other	Change in SO2 (kt)	−27.00	−3.90
10	Modelling	Irz	2017	HIC/MIC	Reduce ASF no substitute	DRCD	% Deaths Avoided	0.40	−0.40	GHG	% CO2 emissions Avoided		−0.90
9	Modelling	Irz	2016	HIC/MIC	'Sustainable Diet'	DRCD	# Deaths Avoided	2331.00	−3.50	Other	Change in SO2 (kt)	−67.00	−9.50
10	Modelling	Irz	2017	HIC/MIC	Substitute ASF with PSF	DRCD	% Deaths Avoided		−3.80	GHG	% CO2 emissions Avoided		−2.80
10	Modelling	Irz	2017	HIC/MIC	Substitute meat with other ASF	DRCD	% Deaths Avoided		−0.40	GHG	% CO2 emissions Avoided		−0.30
11	Modelling	Milner	2015	HIC/MIC	Substitute ASF with PSF	DRCD	# Reduction in YLL (at year 30)	511 348.00	−5.91	GHG	% Reduction GHG		−17.20
11	Modelling	Milner	2015	HIC/MIC	Substitute ASF with PSF	DRCD	# Reduction in YLL (at year 30)	514 093.00	−5.94	GHG	% Reduction GHG		−18.00
11	Modelling	Milner	2015	HIC/MIC	Substitute ASF with PSF	DRCD	# Reduction in YLL (at year 30)	518 627.00	−5.99	GHG	% Reduction GHG		−21.90
11	Modelling	Milner	2015	HIC/MIC	Substitute ASF with PSF	DRCD	# Reduction in YLL (at year 30)	530 696.00	−6.13	GHG	% Reduction GHG		−30.00

(Continued)

(continued).

Study number	Type of study	First author	Year of publication	Region	Final diet category	Health category	Health unit	Health—absolute difference	Health—relative difference	Env outcome	Env unit	Environment—absolute difference	Environment—relative difference
11	Modelling	Milner	2015	HIC/MIC	Substitute ASF with PSF	DRCD	# Reduction in YLL (at year 30)	565 073.00	−6.53	GHG	% Reduction GHG		−40.00
11	Modelling	Milner	2015	HIC/MIC	Substitute ASF with PSF	DRCD	# Reduction in YLL (at year 30)	638 866.00	−7.38	GHG	% Reduction GHG		−50.00
11	Modelling	Milner	2015	HIC/MIC	Substitute ASF with PSF	DRCD	# Reduction in YLL (at year 30)	657 766.00	−7.60	GHG	% Reduction GHG		−60.00
12	Empirical	Rosi	2017	HIC/MIC	Vegan	BMI	kg/m^2	−0.80	−3.62	GHG	g CO2 eq/day (mean)	−1361.00	−34.38
12	Empirical	Rosi	2017	HIC/MIC	Vegan	BMI	kg/m^2	−0.80	−3.62	WU	L/d	−836.00	−26.62
12	Empirical	Rosi	2017	HIC/MIC	Vegetarian	BMI	kg/m^2	−0.20	−0.90	GHG	g CO2 eq/day (mean)	−1623.00	−41.00
12	Empirical	Rosi	2017	HIC/MIC	Vegetarian	BMI	kg/m^2	−0.20	−0.90	WU	L/d	−686.00	−21.84
13	Modelling	Scarborough	2012	HIC/MIC	Substitute ASF with PSF	Cancer	# Deaths Avoided/yr	8236.00	−9.60	GHG	%reduction		−19.00
13	Modelling	Scarborough	2012	HIC/MIC	Substitute ASF with PSF	Cancer	# Deaths Avoided/yr	8236.00	9.60	LU	%reduction		−42.00
13	Modelling	Scarborough	2012	HIC/MIC	Substitute ASF with PSF	Cancer	# Deaths Avoided/yr	2128.00	−2.50	GHG	%reduction		−3.00
13	Modelling	Scarborough	2012	HIC/MIC	Substitute ASF with PSF	Cancer	# Deaths Avoided/yr	2128.00	2.50	LU	%reduction		−4.00
13	Modelling	Scarborough	2012	HIC/MIC	Substitute ASF with PSF	CVD	# Deaths Avoided/yr	28 674.00	20.30	GHG	%reduction		−19.00
13	Modelling	Scarborough	2012	HIC/MIC	Substitute ASF with PSF	CVD	# Deaths Avoided/yr	28 674.00	20.30	LU	%reduction		−42.00
13	Modelling	Scarborough	2012	HIC/MIC	Substitute ASF with PSF	CVD	# Deaths Avoided/yr	7169.00	−5.10	GHG	%reduction		−3.00
13	Modelling	Scarborough	2012	HIC/MIC	Substitute ASF with PSF	CVD	# Deaths Avoided/yr	7169.00	5.10	LU	%reduction		−4.00

(Continued)

(continued).

Study number	Type of study	First author	Year of publication	Region	Final diet category	Health category	Health unit	Health—absolute difference	Health—relative difference	Env outcome	Env unit	Environment—absolute difference	Environment—relative difference
13	Modelling	Scarborough	2012	HIC/MIC	Substitute meat with other ASF	Cancer	# Deaths Avoided/yr	272.00	−0.30	GHG	%reduction		−9.00
13	Modelling	Scarborough	2012	HIC/MIC	Substitute meat with other ASF	Cancer	# Deaths Avoided/yr	272.00	0.30	LU	%reduction		−39.00
13	Modelling	Scarborough	2012	HIC/MIC	Substitute meat with other ASF	CVD	# Deaths Avoided/yr	1727.00	−1.20	GHG	%reduction		−9.00
13	Modelling	Scarborough	2012	HIC/MIC	Substitute meat with other ASF	CVD	# Deaths Avoided/yr	1727.00	1.20	LU	%reduction		−39.00
14	Empirical	Soret	2014	HIC/MIC	Substitute ASF with PSF	All-cause Mortality/Morbidity	# deaths/1000 person-years (95% CI)	−1.13	−16.97	GHG	Change in CO2 eq (Gt)	−0.66	−21.64
14	Empirical	Soret	2014	HIC/MIC	Vegetarian	All-cause Mortality/Morbidity	# deaths/1000 person-years (95% CI)	−1.10	−16.52	GHG	Change in CO2 eq (Gt)	−0.89	−29.18
15	Modelling	Springmann	2016	HIC/MIC	Dietary Guidelines	DRCD	# Deaths avoided (mean, thousands) in 2050	−1649.71	−9.49	GHG	Food related GHG emissions (Gt CO2-eq)	−0.40	−18.18
15	Modelling	Springmann	2016	HIC/MIC	Dietary Guidelines	CVD	# Deaths avoided (mean, thousands) in 2050	−835.47	−22.90	GHG	Food related GHG emissions (Gt CO2-eq)	−0.40	−18.18
15	Modelling	Springmann	2016	HIC/MIC	Dietary Guidelines	CVD	# Deaths avoided (mean, thousands) in 2050	−288.95	−14.09	GHG	Food related GHG emissions (Gt CO2-eq)	−0.40	−18.18
15	Modelling	Springmann	2016	HIC/MIC	Dietary Guidelines	Cancer	# Deaths avoided (mean, thousands) in 2050	−395.69	−9.93	GHG	Food related GHG emissions (Gt CO2-eq)	−0.40	−18.18

(Continued)

(continued).

Study number	Type of study	First author	Year of publication	Region	Final diet category	Health category	Health unit	Health—absolute difference	Health—relative difference	Env outcome	Env unit	Environment—absolute difference	Environment—relative difference
15	Modelling	Springmann	2016	HIC/MIC	Dietary Guidelines	Diabetes	# Deaths avoided (mean, thousands) in 2050	−129.61	−31.65	GHG	Food related GHG emissions (Gt CO2-eq)	−0.40	−18.18
15	Modelling	Springmann	2016	LIC	Dietary Guidelines	DRCD	# Deaths avoided (mean, thousands) in 2050	−4162.33	−6.11	GHG	Food related GHG emissions (Gt CO2-eq)	−2.30	−27.06
15	Modelling	Springmann	2016	LIC	Dietary Guidelines	CVD	# Deaths avoided (mean, thousands) in 2050	−1057.74	−10.16	GHG	Food related GHG emissions (Gt CO2-eq)	−2.30	−27.06
15	Modelling	Springmann	2016	LIC	Dietary Guidelines	CVD	# Deaths avoided (mean, thousands) in 2050	−1262.27	−11.63	GHG	Food related GHG emissions (Gt CO2-eq)	−2.30	−27.06
15	Modelling	Springmann	2016	LIC	Dietary Guidelines	Cancer	# Deaths avoided (mean, thousands) in 2050	−1617.04	−18.25	GHG	Food related GHG emissions (Gt CO2-eq)	−2.30	−27.06
15	Modelling	Springmann	2016	LIC	Dietary Guidelines	Diabetes	# Deaths avoided (mean, thousands) in 2050	−225.28	−10.22	GHG	Food related GHG emissions (Gt CO2-eq)	−2.30	−27.06
15	Modelling	Springmann	2016	HIC/MIC	Vegan	DRCD	# Deaths avoided (mean, thousands) in 2050	−2366.71	−13.61	GHG	Food related GHG emissions (Gt CO2-eq)	−1.80	−81.82

(Continued)

(continued).

Study number	Type of study	First author	Year of publication	Region	Final diet category	Health category	Health unit	Health—absolute difference	Health—relative difference	Env outcome	Env unit	Environment—absolute difference	Environment—relative difference
15	Modelling	Springmann	2016	HIC/MIC	Vegan	CVD	# Deaths avoided (mean, thousands) in 2050	−1185.27	−32.49	GHG	Food related GHG emissions (Gt CO2-eq)	−1.80	−81.82
15	Modelling	Springmann	2016	HIC/MIC	Vegan	CVD	# Deaths avoided (mean, thousands) in 2050	−431.86	−21.06	GHG	Food related GHG emissions (Gt CO2-eq)	−1.80	−81.82
15	Modelling	Springmann	2016	HIC/MIC	Vegan	Cancer	# Deaths avoided (mean, thousands) in 2050	−594.37	−14.92	GHG	Food related GHG emissions (Gt CO2-eq)	−1.80	−81.82
15	Modelling	Springmann	2016	HIC/MIC	Vegan	Diabetes	# Deaths avoided (mean, thousands) in 2050	−155.22	−37.91	GHG	Food related GHG emissions (Gt CO2-eq)	−1.80	−81.82
15	Modelling	Springmann	2016	LIC	Vegan	DRCD	# Deaths avoided (mean, thousands) in 2050	−6428.14	−9.44	GHG	Food related GHG emissions (Gt CO2-eq)	−5.80	−68.24
15	Modelling	Springmann	2016	LIC	Vegan	CVD	# Deaths avoided (mean, thousands) in 2050	−2037.25	−19.56	GHG	Food related GHG emissions (Gt CO2-eq)	−5.80	−68.24
15	Modelling	Springmann	2016	LIC	Vegan	CVD	# Deaths avoided (mean, thousands) in 2050	−1912.56	−17.62	GHG	Food related GHG emissions (Gt CO2-eq)	−5.80	−68.24
15	Modelling	Springmann	2016	LIC	Vegan	Cancer	# Deaths avoided (mean, thousands) in 2050	−2074.30	−23.40	GHG	Food related GHG emissions (Gt CO2-eq)	−5.80	−68.24

(Continued)

(continued).

Study number	Type of study	First author	Year of publication	Region	Final diet category	Health category	Health unit	Health—absolute difference	Health—relative difference	Env outcome	Env unit	Environment—absolute difference	Environment—relative difference
15	Modelling	Springmann	2016	LIC	Vegan	Diabetes	# Deaths avoided (mean, thousands) in 2050	−404.04	−18.32	GHG	Food related GHG emissions (Gt CO2-eq)	−5.80	−68.24
15	Modelling	Springmann	2016	HIC/MIC	Vegetarian	DRCD	# Deaths avoided (mean, thousands) in 2050	−2146.73	−12.35	GHG	Food related GHG emissions (Gt CO2-eq)	−2.00	−90.91
15	Modelling	Springmann	2016	HIC/MIC	Vegetarian	CVD	# Deaths avoided (mean, thousands) in 2050	−1125.83	−30.86	GHG	Food related GHG emissions (Gt CO2-eq)	−2.00	−90.91
15	Modelling	Springmann	2016	HIC/MIC	Vegetarian	CVD	# Deaths avoided (mean, thousands) in 2050	−384.97	−18.77	GHG	Food related GHG emissions (Gt CO2-eq)	−2.00	−90.91
15	Modelling	Springmann	2016	HIC/MIC	Vegetarian	Cancer	# Deaths avoided (mean, thousands) in 2050	−486.77	−12.22	GHG	Food related GHG emissions (Gt CO2-eq)	−2.00	−90.91
15	Modelling	Springmann	2016	HIC/MIC	Vegetarian	Diabetes	# Deaths avoided (mean, thousands) in 2050	−149.16	−36.43	GHG	Food related GHG emissions (Gt CO2-eq)	−2.00	−90.91
15	Modelling	Springmann	2016	LIC	Vegetarian	DRCD	# Deaths avoided (mean, thousands) in 2050	−5806.70	−8.53	GHG	Food related GHG emissions (Gt CO2-eq)	−6.00	−70.59

(Continued)

(continued).

Study number	Type of study	First author	Year of publication	Region	Final diet category	Health category	Health unit	Health—absolute difference	Health—relative difference	Env outcome	Env unit	Environment—absolute difference	Environment—relative difference
15	Modelling	Springmann	2016	LIC	Vegetarian	CVD	# Deaths avoided (mean, thousands) in 2050	−1851.31	−17.78	GHG	Food related GHG emissions (Gt CO2-eq)	−6.00	−70.59
15	Modelling	Springmann	2016	LIC	Vegetarian	CVD	# Deaths avoided (mean, thousands) in 2050	−1740.27	−16.04	GHG	Food related GHG emissions (Gt CO2-eq)	−6.00	−70.59
15	Modelling	Springmann	2016	LIC	Vegetarian	Cancer	# Deaths avoided (mean, thousands) in 2050	−1858.62	−20.97	GHG	Food related GHG emissions (Gt CO2-eq)	−6.00	−70.59
15	Modelling	Springmann	2016	LIC	Vegetarian	Diabetes	# Deaths avoided (mean, thousands) in 2050	−356.51	−16.17	GHG	Food related GHG emissions (Gt CO2-eq)	−6.00	−70.59
16	Modelling	Springmann	2018a	HIC/MIC	Flexitarian	DRCD	Average # Deaths avoided	−9803.00	−24.40	GHG	MtCO2 eq	−3593.00	−56.59

(Continued)

(continued).

Study number	Type of study	First author	Year of publication	Region	Final diet category	Health category	Health unit	Health—absolute difference	Health—relative difference	Env outcome	Env unit	Environment—absolute difference	Environment—relative difference
16	Modelling	Springmann	2018a	LIC	Flexitarian	DRCD	Average # Deaths avoided	−1378.00	−4.99	GHG	MtCO2 eq	−383.00	−38.61
16	Modelling	Springmann	2018a	HIC/MIC	Flexitarian	DRCD	Average # Deaths avoided	−9803.00	−24.40	LU	M km^2	−992.00	−11.59
16	Modelling	Springmann	2018a	LIC	Flexitarian	DRCD	Average # Deaths avoided	−1378.00	−4.99	LU	M km^2	188.00	11.09
16	Modelling	Springmann	2018a	HIC/MIC	Flexitarian	DRCD	Average # Deaths avoided	−9803.00	−24.40	NU	GgN	−17 003.00	−24.10
16	Modelling	Springmann	2018a	LIC	Flexitarian	DRCD	Average # Deaths avoided	−1378.00	−4.99	NU	GgN	−283.00	−4.65
16	Modelling	Springmann	2018a	HIC/MIC	Flexitarian	DRCD	Average # Deaths avoided	−9803.00	−24.40	PU	GgP	−2199.00	−19.78
16	Modelling	Springmann	2018a	LIC	Flexitarian	DRCD	Average # Deaths avoided	−1378.00	−4.99	PU	GgP	81.00	9.34
16	Modelling	Springmann	2018a	HIC/MIC	Flexitarian	DRCD	Average # Deaths avoided	−9803.00	−24.40	WU	km^3	−203.00	−15.01
16	Modelling	Springmann	2018a	LIC	Flexitarian	DRCD	Average # Deaths avoided	−1378.00	−4.99	WU	km^3	42.00	27.27
16	Modelling	Springmann	2018a	HIC/MIC	Pescatarian/ increase fish	DRCD	Average # Deaths avoided	−10 510.00	−26.17	GHG	MtCO2 eq	−4836.00	−76.17
16	Modelling	Springmann	2018a	LIC	Pescatarian/ increase fish	DRCD	Average # Deaths avoided	−1420.00	−5.14	GHG	MtCO2 eq	−696.00	−70.16
16	Modelling	Springmann	2018a	HIC/MIC	Pescatarian/ increase fish	DRCD	Average # Deaths avoided	−10 510.00	−26.17	LU	M km^2	−1233.00	−14.41

(Continued)

(continued).

Study number	Type of study	First author	Year of publication	Region	Final diet category	Health category	Health unit	Health—absolute difference	Health—relative difference	Env outcome	Env unit	Environment—absolute difference	Environment—relative difference
16	Modelling	Springmann	2018a	LIC	Pescatarian/ increase fish	DRCD	Average # Deaths avoided	−1420.00	−5.14	LU	M km^2	150.00	8.85
16	Modelling	Springmann	2018a	HIC/MIC	Pescatarian/ increase fish	DRCD	Average # Deaths avoided	−10 510.00	−26.17	NU	GgN	−17 526.00	−24.84
16	Modelling	Springmann	2018a	LIC	Pescatarian/ increase fish	DRCD	Average # Deaths avoided	−1420.00	−5.14	NU	GgN	−363.00	−5.97
16	Modelling	Springmann	2018a	HIC/MIC	Pescatarian/ increase fish	DRCD	Average # Deaths avoided	−10 510.00	−26.17	PU	GgP	−2259.00	−20.32
16	Modelling	Springmann	2018a	LIC	Pescatarian/ increase fish	DRCD	Average # Deaths avoided	−1420.00	−5.14	PU	GgP	74.00	8.54
16	Modelling	Springmann	2018a	HIC/MIC	Pescatarian/ increase fish	DRCD	Average # Deaths avoided	−10 510.00	−26.17	WU	km^3	−195.00	−14.42
16	Modelling	Springmann	2018a	LIC	Pescatarian/ increase fish	DRCD	Average # Deaths avoided	−1420.00	−5.14	WU	km^3	45.00	29.22
16	Modelling	Springmann	2018a	HIC/MIC	Substitute ASF with PSF	DRCD	Average # Deaths avoided	−2294.00	−5.71	GHG	MtCO2 eq	−1336.00	−21.04
16	Modelling	Springmann	2018a	LIC	Substitute ASF with PSF	DRCD	Average # Deaths avoided	−151.00	−0.55	GHG	MtCO2 eq	−162.00	−16.33
16	Modelling	Springmann	2018a	HIC/MIC	Substitute ASF with PSF	DRCD	Average # Deaths avoided	−2294.00	−5.71	LU	M km^2	−97.00	−1.13
16	Modelling	Springmann	2018a	LIC	Substitute ASF with PSF	DRCD	Average # Deaths avoided	−151.00	−0.55	LU	M km^2	222.00	13.10
16	Modelling	Springmann	2018a	HIC/MIC	Substitute ASF with PSF	DRCD	Average # Deaths avoided	−2294.00	−5.71	NU	GgN	−62.00	−0.09

(Continued)

(continued).

Study number	Type of study	First author	Year of publication	Region	Final diet category	Health category	Health unit	Health—absolute difference	Health—relative difference	Env outcome	Env unit	Environment—absolute difference	Environment—relative difference
16	Modelling	Springmann	2018a	LIC	Substitute ASF with PSF	DRCD	Average # Deaths avoided	−151.00	−0.55	NU	GgN	212.00	3.49
16	Modelling	Springmann	2018a	HIC/MIC	Substitute ASF with PSF	DRCD	Average # Deaths avoided	−2294.00	−5.71	PU	GgP	−37.00	−0.33
16	Modelling	Springmann	2018a	LIC	Substitute ASF with PSF	DRCD	Average # Deaths avoided	−151.00	−0.55	PU	GgP	37.00	4.27
16	Modelling	Springmann	2018a	HIC/MIC	Substitute ASF with PSF	DRCD	Average # Deaths avoided	−2294.00	−5.71	WU	km^3	52.00	3.85
16	Modelling	Springmann	2018a	LIC	Substitute ASF with PSF	DRCD	Average # Deaths avoided	−151.00	−0.55	WU	km^3	13.00	8.44
16	Modelling	Springmann	2018a	HIC/MIC	Substitute ASF with PSF	DRCD	Average # Deaths avoided	−3891.00	−9.69	GHG	MtCO2 eq	−2671.00	−42.07
16	Modelling	Springmann	2018a	LIC	Substitute ASF with PSF	DRCD	Average # Deaths avoided	−257.00	−0.93	GHG	MtCO2 eq	−389.00	−39.21
16	Modelling	Springmann	2018a	HIC/MIC	Substitute ASF with PSF	DRCD	Average # Deaths avoided	−3891.00	−9.69	LU	M km^2	−280.00	−3.27
16	Modelling	Springmann	2018a	LIC	Substitute ASF with PSF	DRCD	Average # Deaths avoided	−257.00	−0.93	LU	M km^2	233.00	13.75
16	Modelling	Springmann	2018a	HIC/MIC	Substitute ASF with PSF	DRCD	Average # Deaths avoided	−3891.00	−9.69	NU	GgN	−747.00	−1.06
16	Modelling	Springmann	2018a	LIC	Substitute ASF with PSF	DRCD	Average # Deaths avoided	−257.00	−0.93	NU	GgN	158.00	2.60
16	Modelling	Springmann	2018a	HIC/MIC	Substitute ASF with PSF	DRCD	Average # Deaths avoided	−3891.00	−9.69	PU	GgP	−158.00	−1.42

(Continued)

(continued).

Study number	Type of study	First author	Year of publication	Region	Final diet category	Health category	Health unit	Health—absolute difference	Health—relative difference	Env outcome	Env unit	Environment—absolute difference	Environment—relative difference
16	Modelling	Springmann	2018a	LIC	Substitute ASF with PSF	DRCD	Average # Deaths avoided	−257.00	−0.93	PU	GgP	33.00	3.81
16	Modelling	Springmann	2018a	HIC/MIC	Substitute ASF with PSF	DRCD	Average # Deaths avoided	−3891.00	−9.69	WU	km^3	102.00	7.54
16	Modelling	Springmann	2018a	LIC	Substitute ASF with PSF	DRCD	Average # Deaths avoided	−257.00	−0.93	WU	km^3	23.00	14.94
16	Modelling	Springmann	2018a	HIC/MIC	Substitute ASF with PSF	DRCD	Average # Deaths avoided	−5266.00	−13.11	GHG	MtCO2 eq	−4007.00	−63.11
16	Modelling	Springmann	2018a	LIC	Substitute ASF with PSF	DRCD	Average # Deaths avoided	−333.00	−1.21	GHG	MtCO2 eq	−616.00	−62.10
16	Modelling	Springmann	2018a	HIC/MIC	Substitute ASF with PSF	DRCD	Average # Deaths avoided	−5266.00	−13.11	LU	M km^2	−463.00	−5.41
16	Modelling	Springmann	2018a	LIC	Substitute ASF with PSF	DRCD	Average # Deaths avoided	−333.00	−1.21	LU	M km^2	245.00	14.45
16	Modelling	Springmann	2018a	HIC/MIC	Substitute ASF with PSF	DRCD	Average # Deaths avoided	−5266.00	−13.11	NU	GgN	−1431.00	−2.03
16	Modelling	Springmann	2018a	LIC	Substitute ASF with PSF	DRCD	Average # Deaths avoided	−333.00	−1.21	NU	GgN	104.00	1.71
16	Modelling	Springmann	2018a	HIC/MIC	Substitute ASF with PSF	DRCD	Average # Deaths avoided	−5266.00	−13.11	PU	GgP	−279.00	−2.51
16	Modelling	Springmann	2018a	LIC	Substitute ASF with PSF	DRCD	Average # Deaths avoided	−333.00	−1.21	PU	GgP	29.00	3.34
16	Modelling	Springmann	2018a	HIC/MIC	Substitute ASF with PSF	DRCD	Average # Deaths avoided	−5266.00	−13.11	WU	km^3	154.00	11.39
16	Modelling	Springmann	2018a	LIC	Substitute ASF with PSF	DRCD	Average # Deaths avoided	−333.00	−1.21	WU	km^3	33.00	21.43

(Continued)

(continued).

Study number	Type of study	First author	Year of publication	Region	Final diet category	Health category	Health unit	Health—absolute difference	Health—relative difference	Env outcome	Env unit	Environment—absolute difference	Environment—relative difference
16	Modelling	Springmann	2018a	HIC/MIC	Substitute ASF with PSF	DRCD	Average # Deaths avoided	−6587.00	−16.40	GHG	MtCO2 eq	−5342.00	−84.14
16	Modelling	Springmann	2018a	LIC	Substitute ASF with PSF	DRCD	Average # Deaths avoided	−405.00	−1.47	GHG	MtCO2 eq	−842.00	−84.88
16	Modelling	Springmann	2018a	HIC/MIC	Substitute ASF with PSF	DRCD	Average # Deaths avoided	−6587.00	−16.40	LU	M km^2	−646.00	−7.55
16	Modelling	Springmann	2018a	LIC	Substitute ASF with PSF	DRCD	Average # Deaths avoided	−405.00	−1.47	LU	M km^2	256.00	15.10
16	Modelling	Springmann	2018a	HIC/MIC	Substitute ASF with PSF	DRCD	Average # Deaths avoided	−6587.00	−16.40	NU	GgN	−2115.00	−3.00
16	Modelling	Springmann	2018a	LIC	Substitute ASF with PSF	DRCD	Average # Deaths avoided	−405.00	−1.47	NU	GgN	51.00	0.84
16	Modelling	Springmann	2018a	HIC/MIC	Substitute ASF with PSF	DRCD	Average # Deaths avoided	−6587.00	−16.40	PU	GgP	−399.00	−3.59
16	Modelling	Springmann	2018a	LIC	Substitute ASF with PSF	DRCD	Average # Deaths avoided	−405.00	−1.47	PU	GgP	25.00	2.88
16	Modelling	Springmann	2018a	HIC/MIC	Substitute ASF with PSF	DRCD	Average # Deaths avoided	−6587.00	−16.40	WU	km^3	204.00	15.09
16	Modelling	Springmann	2018a	LIC	Substitute ASF with PSF	DRCD	Average # Deaths avoided	−405.00	−1.47	WU	km^3	43.00	27.92
16	Modelling	Springmann	2018a	HIC/MIC	Vegan	DRCD	Average # Deaths avoided	−11 353.00	−28.26	GHG	MtCO2 eq	−5516.00	−86.88
16	Modelling	Springmann	2018a	LIC	Vegan	DRCD	Average # Deaths avoided	−1449.00	−5.25	GHG	MtCO2 eq	−857.00	−86.39
16	Modelling	Springmann	2018a	HIC/MIC	Vegan	DRCD	Average # Deaths avoided	−11 353.00	−28.26	LU	M km^2	−1273.00	−14.88
16	Modelling	Springmann	2018a	LIC	Vegan	DRCD	Average # Deaths avoided	−1449.00	−5.25	LU	M km^2	236.00	13.92
16	Modelling	Springmann	2018a	HIC/MIC	Vegan	DRCD	Average # Deaths avoided	−11 353.00	−28.26	NU	GgN	−18 572.00	−26.33

(Continued)

(continued).

Study number	Type of study	First author	Year of publication	Region	Final diet category	Health category	Health unit	Health—absolute difference	Health—relative difference	Env outcome	Env unit	Environment—absolute difference	Environment—relative difference
16	Modelling	Springmann	2018a	LIC	Vegan	DRCD	Average # Deaths avoided	−1449.00	−5.25	NU	GgN	−457.00	−7.52
16	Modelling	Springmann	2018a	HIC/MIC	Vegan	DRCD	Average # Deaths avoided	−11 353.00	−28.26	PU	GgP	−2556.00	−22.99
16	Modelling	Springmann	2018a	LIC	Vegan	DRCD	Average # Deaths avoided	−1449.00	−5.25	PU	GgP	58.00	6.69
16	Modelling	Springmann	2018a	HIC/MIC	Vegan	DRCD	Average # Deaths avoided	−11 353.00	−28.26	WU	km^3	−89.00	−6.58
16	Modelling	Springmann	2018a	LIC	Vegan	DRCD	Average # Deaths avoided	−1449.00	−5.25	WU	km^3	68.00	44.16
16	Modelling	Springmann	2018a	HIC/MIC	Vegetarian	DRCD	Average # Deaths avoided	−9917.00	−24.69	GHG	MtCO2 eq	−4832.00	−76.11
16	Modelling	Springmann	2018a	LIC	Vegetarian	DRCD	Average # Deaths avoided	−1400.00	−5.07	GHG	MtCO2 eq	−693.00	−69.86
16	Modelling	Springmann	2018a	HIC/MIC	Vegetarian	DRCD	Average # Deaths avoided	−9917.00	−24.69	LU	M km^2	−1172.00	−13.70
16	Modelling	Springmann	2018a	LIC	Vegetarian	DRCD	Average # Deaths avoided	−1400.00	−5.07	LU	M km^2	227.00	13.39
16	Modelling	Springmann	2018a	HIC/MIC	Vegetarian	DRCD	Average # Deaths avoided	−9917.00	−24.69	NU	GgN	−18 430.00	−26.12
16	Modelling	Springmann	2018a	LIC	Vegetarian	DRCD	Average # Deaths avoided	−1400.00	−5.07	NU	GgN	−356.00	−5.86

(Continued)

(continued).

Study number	Type of study	First author	Year of publication	Region	Final diet category	Health category	Health unit	Health—absolute difference	Health—relative difference	Env outcome	Env unit	Environment—absolute difference	Environment—relative difference
16	Modelling	Springmann	2018a	HIC/MIC	Vegetarian	DRCD	Average # Deaths avoided	−9917.00	−24.69	PU	GgP	−2422.00	−21.78
16	Modelling	Springmann	2018a	LIC	Vegetarian	DRCD	Average # Deaths avoided	−1400.00	−5.07	PU	GgP	70.00	8.07
16	Modelling	Springmann	2018a	HIC/MIC	Vegetarian	DRCD	Average # Deaths avoided	−9917.00	−24.69	WU	km^3	−167.00	−12.35
16	Modelling	Springmann	2018a	LIC	Vegetarian	DRCD	Average # Deaths avoided	−1400.00	−5.07	WU	km^3	52.00	33.77
17	Modelling	Springmann	2018b	HIC/MIC	Increase PSF	DRCD	Average # Deaths avoided	−56.00	−0.04	GHG	Change in KtCO2 eq	−610.29	−18.37
17	Modelling	Springmann	2018b	HIC/MIC	Reduce ASF no substitute	DRCD	Average # Deaths avoided	−293.00	−0.20	GHG	Change in KtCO2 eq	−475.11	−11.15
17	Modelling	Springmann	2018b	HIC/MIC	Reduce ASF no substitute	DRCD	Average # Deaths avoided	−15.00	−0.01	GHG	Change in KtCO2 eq	0.52	0.20
18	Modelling	Visecchia	2012	HIC/MIC	Substitute ASF with PSF	BMI	% population obese	−2.64	−27.27	GHG	tCO2 eq emissions per year in Italy	−5406000.00	−1.38

ORCID iDs

Alan D Dangour https://orcid.org/0000-0001-6908-1273
Pauline FD Scheelbeek https://orcid.org/0000-0002-6209-2284

References

[1] Mbow H-O P, Reisinger A, Canadell J and O'Brien P 2017 Special report on climate change, desertification, land degradation, sustainable land management, food security, and greenhouse gas fluxes in terrestrial ecosystems (SR2) Ginevra, IPCC (https://www.ipcc.ch/site/assets/uploads/2018/07/sr2_background_report_final.pdf)

[2] United Nations General Assembly 2015 Transforming our world: the 2030 agenda for sustainable development (available at: www.un.org/en/development/desa/population/migration/generalassembly/docs/globalcompact/A_RES_70_1_E.pdf)

[3] Global Nutrition Report 2020 2020 Global nutrition report: action on equity to end malnutrition (available from: https://globalnutritionreport.org/reports/2020-global-nutrition-report/)

[4] Mbow C *et al* 2019 Food security. Climate change and land: an IPCC special report on climate change, desertification, land degradation, sustainable land management, food security and greenhouse gas fluxes in terrestrial ecosystems: IPCC (https://www.ipcc.ch/site/assets/uploads/2019/11/08_Chapter-5.pdf)

[5] Myers S S, Smith M R, Guth S, Golden C D, Vaitla B, Mueller N D, Dangour A D and Huybers P 2017 Climate change and global food systems: potential impacts on food security and undernutrition *Annu. Rev. Public Health* **38** 259–77

[6] Whitmee S *et al* 2015 Safeguarding human health in the Anthropocene epoch: report of the Rockefeller foundation–lancet commission on planetary health *Lancet* **386** 1973–2028

[7] Wollenberg E *et al* 2016 Reducing emissions from agriculture to meet the 2 C target *Glob. Change Biol.* **22** 3859–64

[8] Friel S *et al* 2009 Public health benefits of strategies to reduce greenhouse-gas emissions: food and agriculture *Lancet* **374** 2016–25

[9] Willett W *et al* 2019 Food in the Anthropocene: the EAT–Lancet Commission on healthy diets from sustainable food systems *Lancet* **393** 447–92

[10] Ranganathan J, Vennard D, Waite R, Dumas P, Lipinski B and Searchinger T 2016 Shifting diets for a sustainable food future World Resources Institute (https://files.wri.org/s3fs-public/Shifting_Diets_for_a_Sustainable_Food_Future_1.pdf)

[11] Aleksandrowicz L, Green R, Joy E J, Smith P and Haines A 2016 The impacts of dietary change on greenhouse gas emissions, land use, water use, and health: a systematic review *PloS One* **11** e0165797

[12] Perignon M, Vieux F, Soler L-G, Masset G and Darmon N 2017 Improving diet sustainability through evolution of food choices: review of epidemiological studies on the environmental impact of diets *Nutrition Rev.* **75** 2–17

[13] Patz J A, Stull V J and Limaye V S 2020 A low-carbon future could improve global health and achieve economic benefits *JAMA* **323** 1247–8

[14] Ridoutt B G, Hendrie G A and Noakes M 2017 Dietary strategies to reduce environmental impact: a critical review of the evidence base *Adv. Nutrition* **8** 933–46

[15] Alae-Carew C, Nicoleau S, Bird F A, Hawkins P, Tuomisto H L,, Haines A, Dangour A D and Scheelbeek P F D 2020 The impact of environmental changes on the yield and nutritional quality of fruits, nuts and seeds: a systematic review *Environ. Res. Lett.* **15** 023002

[16] Scheelbeek P F D, Bird F A, Tuomisto H L, Green R, Harris F B, Joy E J M, Chalabi Z, Allen E, Haines A and Dangour A D 2018 Effect of environmental changes on vegetable and legume yields and nutritional quality *Proc. Natl Acad. Sci. USA* **115** 6804–9

[17] Nelson M E, Hamm M W, Hu F B, Abrams S A and Griffin T S 2016 Alignment of healthy dietary patterns and environmental sustainability: a systematic review *Adv. Nutrition* **7** 1005–25

[18] Hallström E, Carlsson-Kanyama A and Börjesson P 2015 Environmental impact of dietary change: a systematic review *J. Clean. Prod.* **91** 1–11

[19] Payne C L, Scarborough P and Cobiac L 2016 Do low-carbon-emission diets lead to higher nutritional quality and positive health outcomes? A systematic review of the literature *Public Health Nutr.* **19** 2654–61

[20] Moher D, Liberati A, Tetzlaff J, Altman D G and Group P 2009 Preferred reporting items for systematic reviews and meta-analyses: the PRISMA statement *PloS Med.* **6** e1000097

[21] Haddaway N, Macura B, Whaley P and Pullin A 2017 ROSES for systematic review protocols Version 1:10.6084 (https://www.roses-reporting.com/systematic-review-protocols)

[22] Jarmul S, Liew Z, Haines A and Scheelbeek P 2019 Climate change mitigation in food systems: the environmental and health impacts of shifting towards sustainable diets, a systematic review protocol *Wellcome Open Res.* **4** 205

[23] Van Voorn G, Verburg R, Kunseler E-M, Vader J and Janssen P H 2016 A checklist for model credibility, salience, and legitimacy to improve information transfer in environmental policy assessments *Environ. Modelling Softw.* **83** 224–36

[24] (CASP) CASP CASP randomized controlled trial checklist 2013 (available at: https://casp-uk.net/wp-content/uploads/2018/01/CASP-Randomised-Controlled-Trial-Checklist-2018.pdf)

[25] World Bank 2020 World Bank Country and lending groups (available at https://datahelpdesk.worldbank.org/knowledgebase/articles/906519-world-bank-country-and-lending-groups#:~:text=For%20the%20current%202021%20fiscal,those%20with%20a%20GNI%20per)

[26] Biesbroek S, Bueno-de-mesquita H B, Peeters P H M, Verschuren W M M, van der Schouw Y T, Kramer G F H, Tyszler M and Temme E H 2014 Reducing our environmental footprint and improving our health: greenhouse gas emission and land use of usual diet and mortality in EPIC-NL: a prospective cohort study *Environ. Health* **13** 27

[27] Irz X, Leroy P, Requillart V and Soler L G 2016 Welfare and sustainability effects of dietary recommendations *Ecol. Econ.* **130** 139–55

[28] Biesbroek S, Verschuren W M M, Boer J M A, van de Kamp M E, van der Schouw Y T, Geelen A, Looman M and Temme E H M 2017 Does a better adherence to dietary guidelines reduce mortality risk and environmental impact in the Dutch sub-cohort of the European Prospective Investigation into Cancer and Nutrition? *Br. J. Nutrition* **118** 69–80

[29] Chen C, Chaudhary A and Mathys A 2019 Dietary change scenarios and implications for environmental, nutrition, human health and economic dimensions of food sustainability *Nutrients* **11** 856

[30] Springmann M, Mason-D'Croz D, Robinson S, Garnett T, Godfray H C J, Gollin D, Rayner M, Ballon P and Scarborough P 2016 Global and regional health effects of future food production under climate change: a modelling study *Lancet* **387** 1937–46

[31] Springmann M, Wiebe K, Mason-D'Croz D, Sulser T B, Rayner M and Scarborough P 2018 Health and nutritional aspects of sustainable diet strategies and their association

with environmental impacts: a global modelling analysis with country-level detail *Lancet Planet. Health* **2** e451–61
[32] Springmann M, Sacks G, Ananthapavan J and Scarborough P 2018 Carbon pricing of food in Australia: an analysis of the health, environmental and public finance impacts *Aust. N.Z. J. Public Health* **42** 523–9
[33] Farchi S, De Sario M, Lapucci E, Davoli M and Michelozzi P 2017 Meat consumption reduction in Italian regions: health co-benefits and decreases in GHG emissions *PloS One* **12** e0182960
[34] Fresan U, Martinez-Gonzalez M A, Sabate J and Bes-Rastrollo M 2019 Global sustainability (health, environment and monetary costs) of three dietary patterns: results from a Spanish cohort (the SUN project) *BMJ Open* **9** e021541
[35] Irz X, Leroy P, Requillart V and Soler L G 2017 Between environmental preservation and health, a cost-benefit analysis of dietary recommendations. Entre preservation de l'environnement et sante, une analyse cout-benefice des recommandations alimentaires pp 1–5 910–2017–199
[36] Salazar T M B, Cai H, Bailey R and Huang J Y 2019 Defining nutritionally and environmentally healthy dietary choices of omega-3 fatty acids *J. Clean. Prod.* **228** 1025–33
[37] Aston L M, Smith J N and Powles J W 2012 Impact of a reduced red and processed meat dietary pattern on disease risks and greenhouse gas emissions in the UK: a modelling study *BMJ Open* **2** e001072
[38] Hobbs D A, Durrant C, Elliott J, Givens D I and Lovegrove J A 2019 Diets containing the highest levels of dairy products are associated with greater eutrophication potential but higher nutrient intakes and lower financial cost in the United Kingdom *Eur. J. Nutrition* **55** 1123–31
[39] Cobiac L J and Scarborough P 2019 Modelling the health co-benefits of sustainable diets in the UK, France, Finland, Italy and Sweden *Eur. J. Clin. Nutr.* **73** 624–33
[40] Milner J, Green R, Dangour A D, Haines A, Chalabi Z, Spadaro J, Markandya A and Wilkinson P 2015 Health effects of adopting low greenhouse gas emission diets in the UK *BMJ Open* **5** e007364
[41] Scarborough P, Allender S, Clarke D, Wickramasinghe K and Rayner M 2012 Modelling the health impact of environmentally sustainable dietary scenarios in the UK *Eur. J. Clin. Nutr.* **66** 710–5
[42] Soret S, Mejia A, Batech M, Jaceldo-Siegl K, Harwatt H and Sabate J 2014 Climate change mitigation and health effects of varied dietary patterns in real-life settings throughout North America *Am. J. Clin. Nutr.* **100** 490S–5S
[43] Viscecchia R, Stasi A and Prosperi M 2012 Health and environmental benefits from combined control of obesity and climate changes *Economia Agro-Alimentare* **14** 75–98
[44] Rosi A *et al* 2017 Environmental impact of omnivorous, ovo-lacto-vegetarian, and vegan diet *Sci. Rep.* **7** 6105
[45] Luo T, Young R and Reig P 2015 Aqueduct projected water stress country rankings. Technical Note (http://www.indiaenvironmentportal.org.in/files/file/aqueduct-water-stress-country-rankings-technical-note.pdf)
[46] Scheelbeek P, Green R, Papier K, Knuppel A, Alae-Carew C, Balkwill A, Key T J, Beral V and Dangour A D 2020 Health impacts and environmental footprints of diets that meet the Eatwell Guide recommendations: analyses of multiple UK studies *BMJ Open* **10** e037554
[47] Rockström J *et al* 2009 Planetary boundaries: exploring the safe operating space for humanity *Ecol. Soc.* **14** 32
[48] Shin H D and Bull R 2019 Three dimensions of design for sustainable behaviour *Sustainability* **11** 4610
[49] Teixeira P J, Carraça E V, Marques M M, Rutter H, Oppert J-M, De Bourdeaudhuij I, Lakerveld J and Brug J 2015 Successful behavior change in obesity interventions in adults: a systematic review of self-regulation mediators *BMC Med.* **13** 84
[50] Dinu M, Pagliai G, Macchi C and Sofi F 2019 Active commuting and multiple health outcomes: a systematic review and meta-analysis *Sports Med.* **49** 437–52
[51] Johansson C, Lövenheim B, Schantz P, Wahlgren L, Almström P, Markstedt A, Strömgren M, Forsberg B and Sommar J N 2017 Impacts on air pollution and health by changing commuting from car to bicycle *Sci. Total Environ.* **584** 55–63
[52] European Public Health Association 2017 Healthy and sustainable diets for European countries (available at https://eupha.org/repository/advocacy/EUPHA_report_on_healthy_and_sustainable_diets_20-05-2017.pdf)
